# Individualized Positive End-Expiratory Pressure and Postoperative Pulmonary Outcomes in Laparoscopic and Robotic Abdominal-Pelvic Surgery: A Systematic Review and Meta-Analysis

**DOI:** 10.7759/cureus.117697

**Published:** 2026-10-09

**Authors:** Noha Salaheldeen Taha Mahde, Husameldin Ali Babiker, Randa Azhari Badawi Abdelmagid, Mehad Abdalla Taha Mohamed, Zawra Omer Ahmed Mohamedsaeed, Asim Ahmed

**Affiliations:** 1 Anesthesiology, College of Medicine, University of Bahri, Khartoum, SDN; 2 Anesthesiology and Intensive Care, University Hospital Limerick, Limerick, IRL; 3 Anesthesiology, Dr. Sulaiman Al Habib Medical Group, Riyadh, SAU; 4 Anesthesiology and Intensive Care, Sudan Medical Specialization Board, Khartoum, SDN; 5 General Practice, Tayma General Hospital, Tayma, SAU; 6 Medicine and Surgery, University of Gezira, Wad Madani, SDN; 7 Research Collaboration Platform, 10 Scholars Academy, Sheridan, USA

**Keywords:** individualized peep, laparoscopic surgery, lung-protective ventilation, positive end-expiratory pressure, postoperative pulmonary complications

## Abstract

Postoperative pulmonary complications remain clinically important after laparoscopic and robot-assisted abdominal-pelvic surgery, where pneumoperitoneum, altered respiratory mechanics, obesity, and procedure-specific positioning can promote lung collapse. This systematic review and meta-analysis evaluated whether individualized intraoperative positive end-expiratory pressure (PEEP), selected using patient-specific physiological or imaging responses, improves postoperative pulmonary outcomes compared with fixed or conventional PEEP. Randomized controlled trials involving adults undergoing eligible minimally invasive abdominal-pelvic procedures were identified through PubMed, Scopus, and Google Scholar and were included when they reported postoperative pulmonary or direct lung-aeration outcomes after extubation. Data were synthesized narratively, with random-effects meta-analysis performed for clinically compatible outcomes, and methodological quality was evaluated using a Cochrane risk-of-bias framework. Across the included evidence, individualized PEEP was generally associated with fewer postoperative pulmonary complications, fewer early postoperative oxygenation problems, and better postoperative lung-aeration measures, whereas binary atelectasis outcomes were more heterogeneous. Favorable findings were observed across driving-pressure-, compliance-, electrical-impedance-tomography-, and lung-ultrasound-guided approaches, although variation in outcome definitions, assessment times, and trial methods limits conclusions regarding the optimal titration strategy. Current evidence suggests that patient-specific PEEP selection may improve postoperative pulmonary outcomes, although larger, standardized trials are needed to determine the most effective titration strategy.

## Introduction and background

Postoperative pulmonary complications (PPCs) remain a major source of perioperative morbidity and include events such as atelectasis, hypoxemia, pulmonary infection, respiratory failure, and the need for postoperative respiratory support. PPCs are associated with greater healthcare resource use, prolonged recovery, and higher postoperative mortality [1]. In a prospective multicenter study of non-cardiothoracic surgical patients, even relatively mild pulmonary complications were associated with greater intensive-care use, longer hospitalization, and increased early mortality [2].

General anesthesia promotes loss of functional residual capacity and dependent lung collapse. Perioperative atelectasis can impair oxygenation and respiratory-system compliance and may contribute to PPCs [3]. These effects are particularly relevant during laparoscopic abdominal-pelvic surgery because carbon-dioxide pneumoperitoneum elevates the diaphragm, increases intra-abdominal and airway pressures, reduces respiratory-system compliance, and can worsen ventilation-perfusion matching [4]. Robot-assisted pelvic surgery may further increase this physiological burden because pneumoperitoneum is often combined with prolonged steep Trendelenburg positioning, which can further reduce lung volume and compliance [5].

The concept of intraoperative lung-protective ventilation developed from evidence that ventilator settings can influence postoperative outcomes even in patients without pre-existing lung injury. In the IMPROVE trial, a protective ventilation strategy incorporating lower tidal volumes, positive end-expiratory pressure (PEEP), and recruitment maneuvers reduced major postoperative complications after abdominal surgery compared with a conventional ventilation strategy [6]. In contrast, the PROVHILO trial found that a strategy using fixed PEEP of 12 cmH₂O with recruitment maneuvers did not reduce PPCs compared with a low-PEEP strategy and was associated with more intraoperative hypotension and vasoactive-drug use [7]. These trials differed in several components of their ventilation strategies and should not be interpreted as showing that PEEP level alone explained the difference in outcomes. Rather, they highlight that applying the same fixed PEEP strategy to all patients may not provide the same physiological benefit in every patient.

This provides a rationale for individualized PEEP selected according to the patient’s physiological response. Driving pressure, which reflects the relationship between tidal volume and respiratory-system compliance, has been associated with postoperative pulmonary risk. An individual-patient-data meta-analysis found that higher intraoperative driving pressure was independently associated with PPCs and that increases in PEEP accompanied by increases in driving pressure were associated with worse pulmonary outcomes [8]. Individualized PEEP can therefore be selected using measures such as minimum driving pressure, maximum static or dynamic compliance, electrical impedance tomography (EIT), a bedside method for assessing regional ventilation, lung ultrasound, esophageal pressure, or other physiological targets intended to balance recruitment against overdistension.

Previous reviews have generally supported individualized PEEP but have differed in scope. A 2022 systematic review and meta-analysis of 10 randomized trials in abdominal surgery reported fewer overall pulmonary complications and postoperative hypoxemia with individualized PEEP, although postoperative atelectasis was not significantly reduced [9]. A 2024 meta-analysis of 30 randomized trials also reported fewer PPCs and better oxygenation and respiratory mechanics, but included both open and laparoscopic abdominal procedures and a broad range of perioperative outcomes [10]. A 2026 systematic review extended the evidence base to minimally invasive thoracic and abdominal surgery and included both randomized and observational studies [11]. These differences in surgical population, study design, outcomes, and comparator strategies leave uncertainty about the specific effect of individualized PEEP in laparoscopic and robot-assisted abdominal-pelvic surgery.

The present review was designed to address this gap by focusing on randomized controlled trials involving adults undergoing laparoscopic or robot-assisted abdominal-pelvic surgery. Eligible studies were required to compare individualized PEEP selected using an explicit patient-specific physiological or imaging method with a fixed, conventional, or standard PEEP strategy and to report at least one postoperative pulmonary or direct lung-aeration outcome after extubation. This more focused framework was intended to provide a direct comparison between patient-specific PEEP selection and standard fixed PEEP while avoiding the broader clinical heterogeneity of previous reviews [9-11].

The primary objective of this systematic review was to determine whether individualized intraoperative PEEP, compared with fixed, conventional, or standard PEEP, reduces PPCs and postoperative atelectasis or loss of lung aeration in adults undergoing laparoscopic or robot-assisted abdominal-pelvic surgery. Secondary objectives were to evaluate postoperative hypoxemia or desaturation, oxygen requirement, pulmonary infection, respiratory failure, reintubation, and other postoperative ventilatory support. We also aimed to describe the methods used to individualize PEEP and, where sufficient data were available, explore important sources of heterogeneity across patient, procedural, and ventilation-related characteristics.

## Review

Methods

Study Design and Reporting

This systematic review of randomized controlled trials was conducted and reported in accordance with the PRISMA 2020 statement [12]. Findings not suitable for quantitative pooling were synthesized using structured narrative principles consistent with SWiM [13]. The review evaluated whether individualized, titrated, or optimized intraoperative PEEP, compared with fixed, conventional, or standard PEEP, improves postoperative pulmonary outcomes after laparoscopic or robot-assisted abdominal-pelvic surgery.

Protocol Registration and Scope Amendment

The literature search was completed on September 5, 2026. A Version 2.0 scope amendment was then finalized on September 6, 2026, before structured outcome extraction, formal Risk of Bias 2 (RoB 2) assessment, effect estimation, or synthesis. The protocol was registered in the SciMENA Scientific Protocol Registry (SciPro-00000235) on September 7, 2026, and the public record displays Version 1.0. The Version 2.0 amendment restricted eligibility to randomized trials of individualized PEEP compared with fixed or conventional PEEP and required at least one qualifying postoperative pulmonary or direct lung-aeration outcome after extubation. This post-search amendment was made to reduce excessive clinical and intervention heterogeneity in the Version 1.0 evidence base and was not based on the direction, magnitude, or statistical significance of the study findings.

Review Question and PICO Framework

The review question was structured using a PICO framework: among adults undergoing laparoscopic or robotic abdominal-pelvic surgery under general anesthesia, does individualized intraoperative PEEP selected using explicit patient-specific physiologic or imaging criteria, compared with fixed or conventional PEEP, reduce PPCs or postoperative loss of lung aeration?

Eligibility and Study-Selection Criteria

Only randomized controlled trials with a concurrent eligible comparator were included. Adults aged 18 years or older undergoing laparoscopic or robot-assisted abdominal-pelvic surgery under general anesthesia with intraoperative mechanical ventilation were eligible. Mixed surgical populations were eligible only when data for the minimally invasive abdominal-pelvic subgroup were separately extractable. Individualized PEEP had to be selected using an explicit patient-specific physiologic or imaging method, including driving-pressure minimization, maximization of static or dynamic respiratory-system compliance, EIT, lung ultrasonography, esophageal pressure, or another clearly defined physiologic titration method. The comparator had to be fixed, conventional, or standard PEEP, or standard ventilation without individualized PEEP titration. At least one qualifying postoperative pulmonary or direct lung-aeration outcome after extubation was required. The complete operational selection criteria are presented in Table 1.

**Table 1:** Study-selection and eligibility criteria. EIT: electrical impedance tomography; PEEP: positive end-expiratory pressure; PPC: postoperative pulmonary complication

Domain	Inclusion criteria	Exclusion criteria
Population	Adults ≥ 18 years undergoing laparoscopic or robot-assisted abdominal-pelvic surgery under general anesthesia with intraoperative mechanical ventilation. Mixed populations were eligible only when the eligible minimally invasive subgroup was separately extractable.	Pediatric populations; open-only surgery; thoracic/thoracoscopic, cardiac, neurosurgical, cesarean, or other non-abdominal-pelvic settings; mixed populations without separable eligible data.
Intervention	Individualized, titrated, or optimized intraoperative PEEP selected using an explicit patient-specific physiologic or imaging method (driving pressure, static/dynamic compliance, EIT, lung ultrasound, esophageal pressure, or comparable explicit method).	Ventilation-mode-only comparisons; recruitment-maneuver-only interventions; tidal-volume-only comparisons; fixed-PEEP-level comparisons without individualized titration; multicomponent bundles in which the individualized-PEEP effect could not be isolated.
Comparator	Fixed, conventional, or standard PEEP, or standard ventilation without individualized PEEP titration. In multiarm trials, at least one eligible individualized-PEEP versus fixed/conventional/standard contrast had to be separately extractable.	No concurrent comparator; individualized/optimized PEEP method versus another individualized/optimized method without an eligible fixed/conventional/standard comparator.
Outcomes	At least one postoperative pulmonary or direct lung-aeration outcome after extubation: atelectasis/loss of aeration, PPC composite/components, postoperative hypoxemia/desaturation, clinically relevant oxygen requirement, pneumonia/pulmonary infection, respiratory failure, reintubation, non-invasive ventilation, high-flow oxygen, or other postoperative ventilatory support.	Only intraoperative oxygenation/mechanics, driving pressure, mechanical power, hemodynamics, diaphragm outcomes, biomarkers, isolated postoperative spirometry, or isolated postoperative PaO₂/FiO₂ without a qualifying clinical pulmonary or direct lung-aeration endpoint.
Study design	Randomized controlled trial with an eligible concurrent comparator and sufficient primary outcome information.	Observational or non-randomized studies, reviews, editorials, case reports, animal/simulation studies, protocol-only reports, or insufficient abstracts.
Report status	Full report in any language when sufficient information could be translated and extracted. Multiple reports from one randomized trial were linked at the trial level.	Retracted reports; duplicate/secondary/companion reports without unique eligible data; reports with insufficient information for eligibility assessment and extraction.

The operational PICO framework is summarized in Table 2.

**Table 2:** PICO framework for the amended Version 2.0 review PICO: Population-Intervention-Comparator-Outcome; EIT: electrical impedance tomography; NIV: non-invasive ventilation; PEEP: positive end-expiratory pressure; PPC: postoperative pulmonary complication

PICO element	Review definition	Operational interpretation
Population	Adults undergoing laparoscopic or robot-assisted abdominal-pelvic surgery under general anesthesia and intraoperative mechanical ventilation.	Included bariatric, colorectal, gynecologic, urologic, and other eligible minimally invasive abdominal-pelvic procedures; mixed populations required separable eligible data.
Intervention	Individualized/titrated/optimized intraoperative PEEP selected using an explicit patient-specific physiologic or imaging criterion.	Driving pressure, static/dynamic compliance, EIT, lung ultrasound, esophageal pressure, or another explicit patient-specific method.
Comparator	Fixed/conventional/standard PEEP or standard intraoperative ventilation without individualized titration.	The individualized-PEEP effect had to be isolated; individualized-versus-individualized comparisons alone were excluded.
Outcomes	Post-extubation pulmonary or direct lung-aeration outcomes.	Primary domains: postoperative atelectasis/loss of aeration and trial-defined PPC composites. Secondary domains: hypoxemia/desaturation, oxygen requirement, pneumonia/infection, respiratory failure, reintubation, NIV/high-flow oxygen, other ventilatory support, and individual PPC components.

Information Sources and Search Strategy

The literature searches were completed on September 5, 2026. MEDLINE via PubMed and Scopus were searched from database inception to that date, and Google Scholar was used as a supplementary discovery source by screening the first 100 results ranked by relevance. No language restriction was applied. The broad Version 1.0 identification search was retained because the Version 2.0 individualized-PEEP question was a strict subset of the broader protective-ventilation domain; eligibility was therefore narrowed during full-text reassessment rather than by replacing the completed identification search. Backward reference-list checking, forward citation searching, and trial-registry checking were used when relevant to identify linked reports or clarify trial identity, protocol features, or outcome reporting. The exact executed search strings and database yields are presented in Table 3.

**Table 3:** Executed literature-search strategies and record yields. Google Scholar was used as a supplementary discovery source, with the first 100 relevance-ranked results screened. The three sources yielded 1,612 records before deduplication.

Database/source	Exact executed search string	Search date	Records identified
PubMed/MEDLINE	(laparoscop*[Title/Abstract] OR robot-assisted[Title/Abstract] OR "robot assisted"[Title/Abstract] OR "robotic surgery"[Title/Abstract] OR pneumoperitoneum[Title/Abstract] OR Trendelenburg[Title/Abstract]) AND ("protective ventilation"[Title/Abstract] OR "lung-protective ventilation"[Title/Abstract] OR "lung protective ventilation"[Title/Abstract] OR "tidal volume"[Title/Abstract] OR PEEP[Title/Abstract] OR "positive end-expiratory pressure"[Title/Abstract] OR "recruitment maneuver"[Title/Abstract] OR "recruitment maneuvers"[Title/Abstract] OR "recruitment manoeuvre"[Title/Abstract] OR "recruitment manoeuvres"[Title/Abstract] OR "open lung"[Title/Abstract] OR "driving pressure"[Title/Abstract] OR "mechanical power"[Title/Abstract] OR "electrical impedance tomography"[Title/Abstract] OR "lung ultrasound"[Title/Abstract]) AND (randomized controlled trial[Publication Type] OR controlled clinical trial[Publication Type] OR random*[Title/Abstract] OR trial[Title/Abstract])	Sep 5, 2026	395
Scopus	TITLE-ABS-KEY(laparoscop* OR "robot-assisted" OR "robot assisted" OR "robotic surgery" OR pneumoperitoneum OR trendelenburg) AND TITLE-ABS-KEY("protective ventilation" OR "lung-protective ventilation" OR "lung protective ventilation" OR "tidal volume" OR peep OR "positive end-expiratory pressure" OR "recruitment maneuver*" OR "recruitment manoeuvre*" OR "open lung" OR "driving pressure" OR "mechanical power" OR "electrical impedance tomography" OR "lung ultrasound") AND TITLE-ABS-KEY(random* OR trial OR controlled)	Sep 5, 2026	1,117
Google Scholar	("protective ventilation" OR "lung protective ventilation" OR PEEP OR "driving pressure" OR "recruitment maneuver") (laparoscopic OR robotic OR pneumoperitoneum) randomized trial - first 100 results ranked by relevance screened.	Sep 5, 2026	100

Record Management, Deduplication, and Study Selection

Search records were merged and deduplicated using DOI, PMID, title, author, publication year, and journal metadata, followed by manual verification of suspected duplicate pairs. Title/abstract screening was followed by full-text assessment against the eligibility criteria. A single principal exclusion reason was recorded for each excluded full-text report according to the predefined hierarchy. For the Version 2.0 re-audit, candidate reports were reassessed sequentially for randomized design, eligible adult minimally invasive abdominal-pelvic population, individualized PEEP as the randomized intervention, an eligible fixed/conventional/standard comparator, at least one qualifying postoperative pulmonary or direct lung-aeration endpoint, and absence of duplicate/companion-report double counting. Multiple reports from the same randomized trial were linked at the trial level. Disagreements were resolved by reviewer discussion and consensus, with third-reviewer adjudication when required.

Data Extraction and Verification

A standardized extraction workbook was used, with trial-level characteristics separated from outcome-level numerical data to prevent duplication across multiple timepoints or definitions. One reviewer performed the initial extraction, and a second reviewer independently verified key eligibility and outcome fields against the source report; discrepancies were resolved by consensus or third-reviewer adjudication. Extraction followed standard systematic-review principles described in the Cochrane Handbook [14] and included study identifiers and design features, participant and procedural characteristics, PEEP-guidance and comparator details, relevant ventilation and co-intervention variables, postoperative outcome definitions and assessment times, arm-level numerical data, effect estimates, and missing-data information.

Special Handling of Multiarm, Mixed-Population, and Companion Trials

For multiarm trials, the principal contrast was the comparison that most directly isolated individualized PEEP against a fixed/conventional comparator. When a recruitment-maneuver-matched fixed-PEEP arm was available, that arm was preferred to a comparator differing simultaneously in both PEEP titration and recruitment strategy. For mixed open and laparoscopic surgical populations, only separately extractable laparoscopic or robotic data were eligible for the main synthesis; pooled open-plus-laparoscopic estimates were not substituted for the eligible subgroup. Potentially overlapping reports were evaluated using authorship, center, procedure, recruitment dates, trial-registration identifiers, sample size, participant flow, and baseline characteristics before being treated as independent cohorts.

Outcome Definition and Synthesis Groups

The primary outcome domains were postoperative atelectasis or loss of lung aeration after extubation and trial-defined composite PPCs. Secondary qualifying outcomes included postoperative hypoxemia/desaturation, clinically relevant oxygen requirement, pulmonary infection/pneumonia, respiratory failure, reintubation, non-invasive ventilation, high-flow oxygen, other postoperative ventilatory support, and individual PPC components. Intraoperative respiratory mechanics and oxygenation were retained only as explanatory variables from otherwise eligible trials. Before pooling, studies were grouped by clinical outcome construct, diagnostic method or measurement scale, and postoperative assessment time. When quantitative pooling was not clinically defensible, results were retained in structured narrative synthesis [13,14].

Statistical Analysis and Meta-Analysis

Quantitative analyses were performed using 10 Scholars App, Version 29. Meta-analysis was undertaken only when at least two trials provided sufficiently compatible populations, individualized-PEEP contrasts, comparators, outcome constructs, measurement approaches, and postoperative timepoints. For dichotomous outcomes, risk ratios (RRs) with 95% confidence intervals (CIs) were calculated from arm-level event data. For continuous outcomes measured on the same scale, mean differences (MDs) with 95% CIs were used. Outcomes measured using materially different constructs or non-equivalent timepoints were not pooled solely to increase study count. Random-effects models were used because true effects were expected to vary across procedures, baseline risk groups, PEEP-selection methods, and comparator strategies. Between-study variance (tau-squared) was estimated using restricted maximum likelihood (REML). Statistical heterogeneity was described using Cochran Q, tau-squared, and I-squared statistics, with clinical compatibility taking precedence over statistical heterogeneity metrics when deciding whether pooling was appropriate [14].

For binary outcomes with a zero event in one study arm, a continuity correction of 0.5 was applied when calculating the study-level RR. Trials with zero events in both arms did not provide an estimable RR and were retained in the qualitative synthesis rather than assigned an artificial treatment effect. Sensitivity analyses were performed when outcome definitions or thresholds materially affected clinical comparability. Funnel plots and regression-based small-study-effect tests were not used because the pooled analyses included too few estimable studies for these methods to be reliable.

Risk-of-Bias Assessment

Risk of bias for outcomes contributing to the principal synthesis was assessed at the result level using the Cochrane RoB 2 framework [15]. The five domains evaluated bias arising from the randomization process, bias due to deviations from intended interventions, bias due to missing outcome data, bias in measurement of the outcome, and bias in selection of the reported result. Domain-level and overall judgments were categorized as low risk, some concerns, or high risk, with written justification for each judgment. Assessments were performed by one reviewer and independently verified by a second reviewer, with disagreements resolved through consensus or third-reviewer adjudication. The overall judgment followed the RoB 2 result-level approach and was not used as an eligibility criterion. Detailed domain tables were retained to support auditability, while the summary traffic-light display followed the robvis visualization convention [16].

Results

Study Selection and PRISMA Flow

The literature search yielded 1,612 records, including 395 from PubMed, 1,117 from Scopus, and the first 100 relevance-ranked records from Google Scholar. After 442 duplicates were removed, 1,170 unique records remained for title and abstract screening. Of these, 988 were excluded, and the remaining 182 reports were sought for full-text retrieval.

Of the 182 reports sought, 18 could not be retrieved after reasonable electronic retrieval attempts, leaving 164 full-text reports for eligibility assessment. Following application of the final Version 2.0 eligibility criteria, 141 reports were excluded. The principal exclusion categories were intervention or randomized contrast outside the refined individualized-PEEP scope (n = 116), no qualifying postoperative pulmonary or direct lung-aeration outcome (n = 10), wrong comparator or no concurrent eligible comparator (n = 6), wrong population or surgical setting (n = 3), duplicate or companion report without unique eligible data (n = 3), non-randomized study design (n = 1), no eligible pulmonary outcome under the original broad criteria (n = 1), and retraction (n = 1).

Under the original broad Version 1.0 framework, 152 unique studies had been considered eligible during evidence mapping. The initial Version 2.0 refinement retained 27 candidate randomized studies; a subsequent comparator audit excluded four trials because both randomized groups received individualized or optimized PEEP without a fixed, conventional, or standard comparator. The final systematic review therefore included 23 unique randomized controlled trials. The complete selection process is shown in Figure 1.

**Figure 1:**
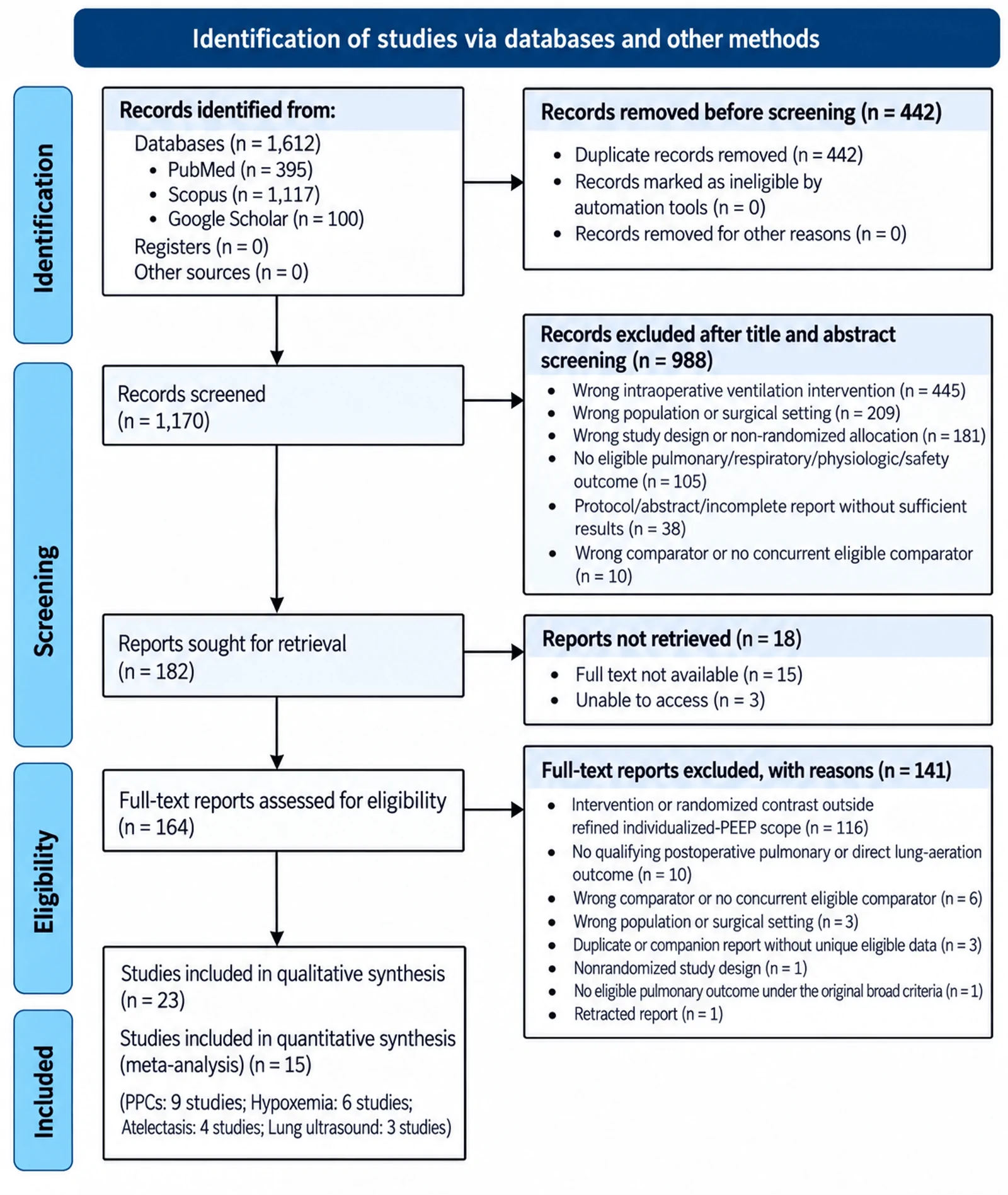
PRISMA 2020 flow diagram for study identification, screening, full-text assessment, and final inclusion. PRISMA 2020 flow diagram illustrating the identification, screening, eligibility assessment, and final inclusion of studies evaluating individualized/optimized positive end-expiratory pressure (PEEP) in laparoscopic and robotic abdominal-pelvic surgery. Reasons for exclusion are reported at both the title/abstract screening and full-text assessment stages. Detailed database search logs and screening records are available from the authors upon reasonable request.

Study Characteristics

The 23 included randomized controlled trials [17-39] were published between 2018 and 2026 and randomized 1,796 participants, of whom 1,743 were included in the reported analyses across all randomized arms. These totals include participants assigned to additional arms in multiarm trials and participants from mixed surgical populations; therefore, the effective sample contributing to each eligible individualized-PEEP versus fixed-PEEP comparison varied by outcome.

The evidence base was geographically concentrated in East Asia. Eleven trials were conducted in China [17-19,21,22,24,30,32,34,37,38], four in South Korea [25,27,31,33], two in Brazil [20,36], two in Egypt [23,35], and two in Turkey [29,39]; one trial each was conducted in India [26] and Belgium [28]. Most were single-center studies or reported a single-center recruitment structure.

Surgical populations were diverse but remained within the minimally invasive abdominal-pelvic scope. Six trials involved laparoscopic bariatric surgery [17,23,28,29,35,36], four focused on laparoscopic colorectal or rectal cancer surgery [18,21,24,32], and five predominantly evaluated robot-assisted radical prostatectomy [19,25,30,37,38]. The remaining studies involved laparoscopic gynecological surgery [26], mixed laparoscopic or robotic lower abdominal/pelvic surgery [27], robot-assisted laparoscopic surgery in steep Trendelenburg [31,39], urologic surgery in the lateral decubitus position [33], or a broader laparoscopic abdominal-pelvic population [34]. Seven trials specifically focused on older adults [18,21,22,24,32,37,38], while six were conducted in bariatric or markedly obese populations [17,23,28,29,35,36].

PEEP individualization methods varied substantially. Driving-pressure-guided titration was the most common strategy and was used in 11 trials [21,22,27,30,31,34-39]. Six trials individualized PEEP according to static or dynamic respiratory-system compliance [17,25,26,28,29,32], three used EIT [19,20,33], and three used lung-ultrasound guidance [18,23,24]. The common comparison was patient-specific PEEP selection rather than assignment of the same PEEP to all participants.

The comparator was more homogeneous. Fixed PEEP of 5 cmH₂O was used in 13 trials [18,19,21,24,26,27,30,31,33-35,37,38], while other trials used fixed PEEP values of 4, 6, 7, 8, or 10 cmH₂O. Where achieved individualized PEEP was reported, values commonly exceeded the fixed comparator and were influenced by obesity, pneumoperitoneum, surgical position, and titration method. Several protocols incorporated recruitment maneuvers, whereas others deliberately avoided them.

Multiarm and mixed-population trials required predefined handling. In Zhang et al. [21], the principal eligible comparison was individualized PEEP versus fixed PEEP 5 cmH₂O, with the zero-PEEP arm treated as secondary. In Silveira et al. [36], the recruitment-matched individualized-PEEP versus fixed-PEEP comparison was prioritized to isolate the PEEP-titration effect. Pereira et al. [20] enrolled both open and laparoscopic abdominal surgery patients; only the separately identifiable laparoscopic subgroup was considered eligible for the main review, and the pooled open-plus-laparoscopic postoperative computed tomography (CT) estimate was not substituted for the eligible subgroup effect.

Postoperative outcomes also varied. Direct assessment of atelectasis or lung aeration by CT or lung ultrasound was the most frequent outcome class [17,18,20,22,24-26,29,31,32,34,38,39]. Other studies primarily evaluated composite PPCs [17,18,24,27,30,33,34,36,37,39], postoperative hypoxemia or oxygen requirement [19,23,26-28,30,35,39], or pulmonary infection scores [21]. Assessment times ranged from the postanesthesia care unit (PACU) or first hour after extubation to postoperative days (PODs) 1-7 or hospital discharge. The complete study-level extraction is presented in Table 4.

**Table 4:** Study characteristics and extraction summary for the 23 included randomized controlled trials. Study labels include the manuscript reference number in the same column as the study identifier [17-39]. EIT: electrical impedance tomography; PEEP: positive end-expiratory pressure; PPC: postoperative pulmonary complication; CT: computed tomography; LUS: lung-ultrasound score; PACU: postanesthesia care unit; POD: postoperative day

Study ID/citation	Country	Randomized/analyzed N	Procedure/population	Approach/position	PEEP guidance	Achieved PEEP	Comparator	Main postoperative outcome/timing
Li et al. [17] (U0002)	China	40/40	Laparoscopic bariatric surgery; adults with obesity	Laparoscopic	Dynamic-compliance-guided individualized PEEP	Median 15 (13-17) cmH₂O; range 8-19	Fixed PEEP 8 cmH₂O	CT-defined postoperative atelectasis; PPCs; CT 60-90 min after extubation; PPC follow-up
Feng et al. [18] (U0014)	China	40/40	Laparoscopic radical rectal cancer surgery; age > 65 y	Laparoscopic; Trendelenburg/pneumoperitoneum	Lung-ultrasound-guided individualized PEEP	Median 11 (7-11.75) cmH₂O	Fixed PEEP 5 cmH₂O	Postoperative atelectasis; PPCs; POD1; postoperative follow-up
Pan et al. [19] (U0015)	China	56/52	Robot-assisted laparoscopic radical prostatectomy	Robotic/laparoscopic; steep Trendelenburg	EIT-guided individualized PEEP	Median 16 cmH₂O (range 12-18)	Fixed PEEP 5 cmH₂O	PACU postoperative hypoxemia; PACU
Pereira et al. [20] (U0022)	Brazil	40/40	Mixed open/laparoscopic abdominal surgery; eligible laparoscopic subgroup only	Laparoscopic subgroup	EIT-guided individualized PEEP	Median 12 cmH₂O; range 6-16	Fixed PEEP 4 cmH₂O	CT-defined atelectasis after extubation; early postoperative CT; subgroup result graph-only
Zhang et al. [21] (U0054)	China	90/90	Laparoscopic radical colorectal cancer surgery; age > 65 y	Laparoscopic	Driving-pressure/esophageal-manometry-guided individualized PEEP	10.8 ± 1.4 cmH₂O	Fixed PEEP 5 cmH₂O; PEEP 0 third arm secondary	Clinical Pulmonary Infection Score; POD1
Xu et al. [22] (U0087)	China	49/49	Laparoscopic surgery in elderly adults	Laparoscopic; pneumoperitoneum	Driving-pressure-guided individualized PEEP	Patient-specific; approximately 4-7 cmH₂O across stages	Fixed PEEP 6 cmH₂O	PACU lung aeration/atelectasis; PPCs; 15 min in PACU; PPC follow-up
Elshazly et al. [23] (U0096)	Egypt	40/40	Laparoscopic bariatric surgery; BMI > 35 kg/m²	Laparoscopic	Lung-ultrasound-guided individualized PEEP	Not reported	Fixed PEEP 4 cmH₂O	Early postoperative hypoxia/basal collapse and other PPCs; within the first 24 h
Ma et al. [24] (U0120)	China	80/73	Laparoscopic colorectal cancer surgery in elderly adults	Laparoscopic; Trendelenburg	Lung-ultrasound-guided individualized PEEP	Median 9 (7-11) cmH₂O	Fixed PEEP 5 cmH₂O	Atelectasis severity; PPCs; hypoxemia; POD1/POD3; outcomes within 7 d
Yoon et al. [25] (U0143)	South Korea	60/60	Robot-assisted radical prostatectomy	Robotic/laparoscopic; steep Trendelenburg	Compliance-based individualized PEEP	Not reported	Fixed PEEP 7 cmH₂O	Post-extubation lung-ultrasound atelectasis; respiratory complications; immediately after extubation
Deeparaj et al. [26] (U0148)	India	82/82	Laparoscopic gynecological surgery	Laparoscopic	Compliance-based optimum PEEP	Median 16 (14-18) cmH₂O	Fixed PEEP 5 cmH₂O	LUS atelectasis; oxygen requirement; 30 min after extubation/PACU; oxygen through 24 h
Kim et al. [27] (U0158)	South Korea	384/363	Laparoscopic or robotic lower abdominal/pelvic surgery	Laparoscopic/robotic	Driving-pressure-guided individualized PEEP	13.6 ± 2.1 cmH₂O	Fixed PEEP 5 cmH₂O	Composite PPCs; desaturation-related complications; within 7 d
Van Hecke et al. [28] (U0210)	Belgium	100/100	Laparoscopic bariatric surgery	Laparoscopic	Dynamic-compliance-guided individualized PEEP	Not reported	Fixed PEEP 10 cmH₂O	Postoperative hypoxemia; first 2 PODs
Göçmen et al. [29] (U0292)	Turkey	70/70	Laparoscopic bariatric surgery; BMI ≥ 35 kg/m²	Laparoscopic	Dynamic-compliance-guided individualized PEEP	Not reported	Fixed PEEP 8 cmH₂O	PACU lung aeration loss; postoperative oxygenation; 30 min in PACU
Luo et al. [30] (U0293)	China	42/42	Robot-assisted laparoscopic radical prostatectomy	Robotic/laparoscopic; steep Trendelenburg	Driving-pressure-guided individualized PEEP	11.29 ± 1.01 cmH₂O during pneumoperitoneum/Trendelenburg; 7.05 ± 1.20 after deflation	Fixed PEEP 5 cmH₂O	PPCs; post-extubation hypoxemia; within 7 d
Cho et al. [31] (U0295)	South Korea	63/63	Robot-assisted laparoscopic surgery	Robotic/laparoscopic; steep Trendelenburg	Driving-pressure-guided individualized PEEP	Median ~8 (8-9) cmH₂O during Trendelenburg	Fixed PEEP 5 cmH₂O	Recovery lung-ultrasound de-aeration; PPCs; 30 min in PACU; postoperative
Li et al. [32] (U0305)	China	70/64	Laparoscopic rectal cancer surgery; age 65-75 y	Laparoscopic; Trendelenburg	Static-compliance-guided individualized PEEP	Not reported	Fixed PEEP 4 cmH₂O	PACU lung-ultrasound atelectasis score; before PACU discharge
Kim et al. [33] (U0323)	South Korea	74/71	Laparoscopic/robotic urologic surgery	Laparoscopic/robotic; lateral decubitus	EIT-guided individualized PEEP	Not reported	Fixed PEEP 5 cmH₂O	PPCs until discharge; until discharge
Zhang et al. [34] (U0327)	China	106/102	Laparoscopic abdominal-pelvic surgery; medium/high pulmonary risk	Laparoscopic; Trendelenburg/pneumoperitoneum	Driving-pressure-guided individualized PEEP	Median 7 (6-7) cmH₂O	Fixed PEEP 5 cmH₂O	Post-extubation/POD1 LUS; atelectasis; PPCs; 15 min after extubation, POD1, and within 7 d
Elbehairy et al. [35] (U0386)	Egypt	60/60	Laparoscopic bariatric surgery; BMI 40-50 kg/m²	Laparoscopic	Driving-pressure-guided individualized PEEP	Not reported	Fixed PEEP 5 cmH₂O	Postoperative hypoxemia requiring oxygen; Post-extubation/postoperative
Silveira et al. [36] (U0389)	Brazil	78/76	Laparoscopic bariatric surgery	Laparoscopic	Driving-pressure-guided PEEP titration	15.4 ± 3.3 cmH₂O during pneumoperitoneum in individualized arm	Recruitment-matched fixed PEEP 6 cmH₂O	PPCs; postoperative oxygen requirement; postoperative follow-up
Zhu et al. [37] (U0540)	China	46/46	Robot-assisted radical prostatectomy; age 65-80 y; ARISCAT > 26	Robotic/laparoscopic; Trendelenburg	Driving-pressure-guided individualized PEEP	Not reported	Fixed PEEP 5 cmH₂O	PPCs; within 7 d
Wang et al. [38] (U0845)	China	50/50	Robot-assisted radical prostatectomy; age 65-80 y	Robotic/laparoscopic; Trendelenburg	Driving-pressure-guided individualized PEEP	Not reported	Fixed PEEP 5 cmH₂O	PACU atelectatic aeration loss; PPCs; PACU; within 3 d
Uysal et al. [39] (U1160)	Turkey	76/70	Robot-assisted laparoscopic surgery	Robotic/laparoscopic; steep Trendelenburg	Driving-pressure-guided personalized PEEP	Not reported	Fixed PEEP 4 cmH₂O	Postoperative LUS, oxygenation, and PPCs; first postoperative hour; oxygenation through 48 h

Key Numerical Findings

Postoperative lung aeration was assessed using different modalities and at different postoperative timepoints, so these findings were interpreted separately rather than as directly equivalent measures. Li et al. [17] used CT and reported atelectatic lung tissue of 9.5% ± 4.3% versus 13.1% ± 5.3% at 60-90 minutes after extubation (MD approximately −3.7 percentage points, 95% CI −6.8 to −0.5; P = 0.025). Lung-ultrasound-based assessments also generally favored individualized PEEP. Feng et al. [18] observed no postoperative atelectasis events in the individualized group versus 5/20 controls on POD1, accompanied by a lower lung-ultrasound score (LUS), while Ma et al. [24] reported fewer atelectasis events within seven days (15/37 vs. 27/36) and a lower POD1 LUS.

Continuous LUS findings were favorable across several early postoperative assessments, although the exact timing and scoring approaches differed between studies. Yoon et al. [25] reported 8.1 ± 5.7 versus 12.2 ± 4.2 immediately after extubation; Li et al. [32] reported 10.3 ± 4.6 versus 14.4 ± 4.7 before PACU discharge; and Uysal et al. [39] reported 11.2 ± 2.2 versus 13.1 ± 3.2 during the first postoperative hour. Göçmen et al. [29] reported modified LUS values of 2.20 ± 1.16 versus 5.80 ± 2.14 in the PACU. Deeparaj et al. [26] and Zhang et al. [34] also reported lower postoperative LUS with individualized PEEP, although their principal LUS summaries were reported as medians and interquartile ranges.

Not all dichotomized atelectasis definitions separated the groups. Cho et al. [31] reported lower continuous PACU LUS with individualized PEEP but very high rates of the study-defined significant atelectasis threshold in both groups (29/31 vs. 32/32). This discrepancy supported separate interpretation of continuous aeration burden and binary threshold outcomes. For Pereira et al. [20], the eligible laparoscopic subgroup postoperative CT result was graphically presented but not available as a sufficiently precise arm-level mean and standard deviation; the pooled open-plus-laparoscopic estimate was not used.

Trial-defined PPC composites generally favored individualized PEEP. Feng et al. [18] reported PPCs in 1/20 versus 7/20 participants; Ma et al. [24], 18/37 versus 28/36; Zhang et al. [34], 7/51 versus 16/51; and Silveira et al. [36], 3/26 versus 8/25 in the recruitment-matched comparison. The largest trial, Kim et al. [27], reported 25/178 versus 36/185. Kim et al. [33] reported 4/35 versus 7/36, and Uysal et al. [39] reported 9/35 versus 12/35. Li et al. [17] reported 4/20 in each group, Luo et al. [30] reported two postoperative pulmonary events in each arm, and Zhu et al. [37] reported no PPCs in either group.

Postoperative oxygenation outcomes were related but not directly equivalent across studies because definitions and assessment periods varied. For hypoxemia and desaturation events, Pan et al. [19] reported PACU hypoxemia in 1/26 versus 7/26 participants, Kim et al. [27] reported desaturation-related complications in 8/178 versus 30/185 (RR 0.28, 95% CI 0.13-0.59), Ma et al. [24] reported 17/37 versus 23/36 hypoxemia events, and Luo et al. [30] reported 2/21 in both groups. Oxygen-support outcomes were reported separately: Elbehairy et al. [35] observed postoperative hypoxia requiring supplemental oxygen in 0/30 versus 7/30, while Deeparaj et al. [26] reported a lower PACU oxygen requirement (30/41 vs. 39/41) and a shorter duration of oxygen supplementation. Overall, the direction of these oxygenation-related findings generally favored individualized PEEP, despite differences in endpoint definitions and postoperative timing.

Van Hecke et al. [28] reported very low hypoxemia incidence in both groups (2.1% vs. 1.3%; P = 0.264). Because these published percentages were not compatible with integer event counts based on the reported group denominators, they were retained as reported rather than converted into artificial event counts. Uysal et al. [39] additionally reported improved first-hour PaO₂ (104.6 ± 27.4 vs. 85.6 ± 27.4 mmHg; P = 0.005), whereas later oxygenation differences at 24 and 48 hours were not significant.

Zhang et al. [21] reported a lower postoperative Clinical Pulmonary Infection Score on POD1 with individualized PEEP (2.8 ± 1.4 vs. 3.5 ± 1.5; P < 0.05). Severe postoperative respiratory events such as pneumonia, reintubation, prolonged invasive ventilation, or pneumothorax were uncommon when reported and were consequently imprecise at the individual-trial level. The complete numerical extraction is presented in Table 5.

**Table 5:** Key postoperative numerical findings extracted from the included randomized trials. CT: computed tomography; MD: mean difference; CI: confidence interval; NA: not applicable; NR: not reported or not extractable from the published report; PPC: postoperative pulmonary complication; POD: postoperative day; IQR: interquartile range; PACU: postanesthesia care unit; LUS: lung-ultrasound score; PEEP: positive end-expiratory pressure; RR: risk ratio

Study ID/citation	Outcome domain	Outcome/timepoint	N, individualized	N, comparator	Events, individualized	Events, comparator	Continuous result, individualized	Continuous result, comparator	Reported comparison	Synthesis status
Li et al. [17] (U0002)	Atelectasis/lung aeration	CT atelectatic lung tissue, 60-90 min after extubation	19	19	NA	NA	9.5% ± 4.3%	13.1% ± 5.3%	MD −3.7 percentage points (95% CI −6.8 to −0.5); P = 0.025	Poolable continuous CT only if compatible trial exists
Li et al. [17] (U0002)	PPCs	Trial-reported PPCs	20	20	4	4	NA	NA	No between-group difference	Poolable binary PPC
Feng et al. [18] (U0014)	Atelectasis	Postoperative atelectasis, POD1	20	20	0	5	NA	NA	P = 0.047	Poolable binary atelectasis
Feng et al. [18] (U0014)	Lung aeration	LUS, POD1	20	20	NA	NA	8.5 (6-9.75)	12.5 (10-13.75)	P < 0.001	Narrative; median/IQR
Feng et al. [18] (U0014)	PPCs	Any trial-defined PPC	20	20	1	7	NA	NA	P = 0.044	Poolable binary PPC
Pan et al. [19] (U0015)	Hypoxemia	PACU postoperative hypoxemia	26	26	1	7	NA	NA	P = 0.021	Poolable binary hypoxemia
Pereira et al. [20] (U0022)	Atelectasis	Eligible laparoscopic subgroup CT after extubation	NR	NR	NR	NR	NR	NR	Subgroup result graph-only; pooled open+lap result not used	Narrative unless prespecified digitization
Zhang et al. [21] (U0054)	Pulmonary infection	Clinical Pulmonary Infection Score, POD1	30	30	NA	NA	2.8 ± 1.4	3.5 ± 1.5	P < 0.05	Single-study continuous outcome
Xu et al. [22] (U0087)	Lung aeration	PACU 12-zone LUS, 15 min	17	16	NA	NA	NR	NR	Individualized PEEP significantly lower; exact arm-level values figure-only; P < 0.05	Narrative unless graph digitization
Elshazly et al. [23] (U0096)	Hypoxia/collapse	Early postoperative hypoxia/basal collapse	20	20	0	5	NA	NA	P = 0.047	Clinically related binary event
Ma et al. [24] (U0120)	PPCs	Any PPC within 7 d	37	36	18	28	NA	NA	RR 0.625 (95% CI 0.430-0.909); P = 0.010	Poolable binary PPC
Ma et al. [24] (U0120)	Atelectasis	Atelectasis within 7 d	37	36	15	27	NA	NA	RR 0.541 (95% CI 0.350-0.834); P = 0.003	Poolable binary atelectasis
Ma et al. [24] (U0120)	Hypoxemia	Hypoxemia within 7 d	37	36	17	23	NA	NA	RR 0.719 (95% CI 0.469-1.102); P = 0.124	Poolable binary hypoxemia
Ma et al. [24] (U0120)	Lung aeration	LUS, POD1	37	36	NA	NA	4 (1-5)	6 (4-10)	P = 0.001	Narrative; median/IQR
Yoon et al. [25] (U0143)	Lung aeration	12-zone LUS immediately after extubation	30	30	NA	NA	8.1 ± 5.7	12.2 ± 4.2	MD −4.13 (95% CI −6.74 to −1.53); P = 0.002	Poolable early total LUS
Deeparaj et al. [26] (U0148)	Lung aeration	Modified LUS, 30 min after extubation	41	41	NA	NA	1 (1-2)	3 (2-3)	P = 0.0001	Narrative; median/IQR
Deeparaj et al. [26] (U0148)	Oxygen requirement	Supplemental oxygen in PACU	41	41	30	39	NA	NA	P = 0.007	Poolable binary oxygen requirement
Deeparaj et al. [26] (U0148)	Oxygen requirement	Duration of supplemental oxygen, first 24 h	41	41	NA	NA	10 (0-15) min	25 (20-30) min	P < 0.0001	Narrative; median/IQR
Kim et al. [27] (U0158)	PPCs	Composite PPCs within 7 d	178	185	25	36	NA	NA	RR 0.72 (95% CI 0.45-1.15); P = 0.215	Poolable binary PPC
Kim et al. [27] (U0158)	Desaturation	Desaturation-related pulmonary complication	178	185	8	30	NA	NA	RR 0.28 (95% CI 0.13-0.59); P = 0.001	Poolable binary hypoxemia/desaturation
Van Hecke et al. [28] (U0210)	Hypoxemia	SpO₂ < 90% during first 2 PODs	50	50	NR	NR	NA	NA	Published incidence 2.1% vs. 1.3%; P = 0.264	Narrative; percentages not convertible to integer events
Göçmen et al. [29] (U0292)	Lung aeration	Modified LUS, 30 min in PACU	35	35	NA	NA	2.20 ± 1.16	5.80 ± 2.14	P < 0.001	Continuous scale differs from total LUS set
Göçmen et al. [29] (U0292)	Oxygenation	PaO₂/FiO₂ in PACU	35	35	NA	NA	425.11 ± 127.13	311.65 ± 92.59	P < 0.001	Narrative/secondary continuous oxygenation
Luo et al. [30] (U0293)	PPC/hypoxemia	Only postoperative pulmonary event: hypoxemia	21	21	2	2	NA	NA	No significant difference	Poolable binary PPC/hypoxemia as prespecified domain
Cho et al. [31] (U0295)	Lung aeration	PACU LUS, 30 min	31	32	NA	NA	9 (7.5-12)	13.5 (11-15)	P < 0.001	Narrative; median/IQR
Cho et al. [31] (U0295)	Atelectasis	Study-defined significant atelectasis	31	32	29	32	NA	NA	P = 0.458	Poolable binary atelectasis; threshold sensitivity issue
Li et al. [32] (U0305)	Lung aeration	LUS before PACU discharge	32	32	NA	NA	10.3 ± 4.6	14.4 ± 4.7	MD −4.06 (95% CI −6.3 to −1.7); P < 0.01	Poolable early total LUS
Kim et al. [33] (U0323)	PPCs	Any PPC until discharge	35	36	4	7	NA	NA	P = 0.545	Poolable binary PPC
Zhang et al. [34] (U0327)	Lung aeration	LUS, POD1	51	51	NA	NA	4 (3-6)	6 (5-8)	P < 0.001	Narrative; median/IQR
Zhang et al. [34] (U0327)	Atelectasis	Ultrasound-detected atelectasis, 15 min after extubation	51	51	30	41	NA	NA	P=0.018	Poolable binary atelectasis
Zhang et al. [34] (U0327)	PPCs	Any PPC within 7 d	51	51	7	16	NA	NA	P = 0.033	Poolable binary PPC
Zhang et al. [34] (U0327)	Atelectasis	CT-identified atelectasis component within 7 d	51	51	5	13	NA	NA	P = 0.038	Secondary binary atelectasis component
Elbehairy et al. [35] (U0386)	Hypoxemia	Postoperative hypoxia requiring supplemental oxygen	30	30	0	7	NA	NA	0% vs. 23.3%	Poolable binary hypoxemia
Silveira et al. [36] (U0389)	PPCs	Recruitment-matched titrated vs. fixed-PEEP contrast	26	25	3	8	NA	NA	No significant individual difference	Poolable binary PPC
Zhu et al. [37] (U0540)	PPCs	PPCs within 7 d	23	23	0	0	NA	NA	No events in either arm	Double-zero; narrative, not estimable in RR model
Wang et al. [38] (U0845)	Atelectasis/PPCs	PACU aeration loss/PPCs within 3 d	25	25	NR	NR	NR	NR	Numerical postoperative outcome data were not reported in an extractable form	Narrative only
Uysal et al. [39] (U1160)	Lung aeration	Total LUS, first postoperative hour	35	35	NA	NA	11.2 ± 2.2	13.1 ± 3.2	P = 0.007	Poolable early total LUS
Uysal et al. [39] (U1160)	Oxygenation	Arterial PaO₂, first postoperative hour	35	35	NA	NA	104.6 ± 27.4	85.6 ± 27.4	P = 0.005	Narrative/secondary continuous oxygenation
Uysal et al. [39] (U1160)	PPCs	Any PPC	35	35	9	12	NA	NA	25.7% vs. 34.3%; P > 0.05	Poolable binary PPC

Meta-Analysis

Random-effects meta-analysis was performed only where at least two trials provided clinically compatible postoperative outcomes and defensible arm-level numerical data. Heterogeneity variance was estimated using the REML approach.

Nine trials provided estimable binary data for trial-defined PPCs [17,18,24,27,30,33,34,36,39], contributing 852 participants to the pooled comparison. PPCs occurred in 73/423 participants receiving individualized PEEP and 120/429 receiving fixed or conventional PEEP. Individualized PEEP was associated with fewer PPCs (RR 0.63, 95% CI 0.50-0.80; P < 0.001), with no observed statistical heterogeneity (I² = 0%; τ² ≈ 0; Q = 5.06, df = 8, P = 0.751). This absence of detected statistical heterogeneity does not exclude clinical heterogeneity, as PPC definitions and trial characteristics varied across studies. Zhu et al. [37] reported zero events in both groups and therefore contributed no finite RR information to the inverse-variance pooled effect. The PPC forest plot is shown in Figure 2.

**Figure 2:**
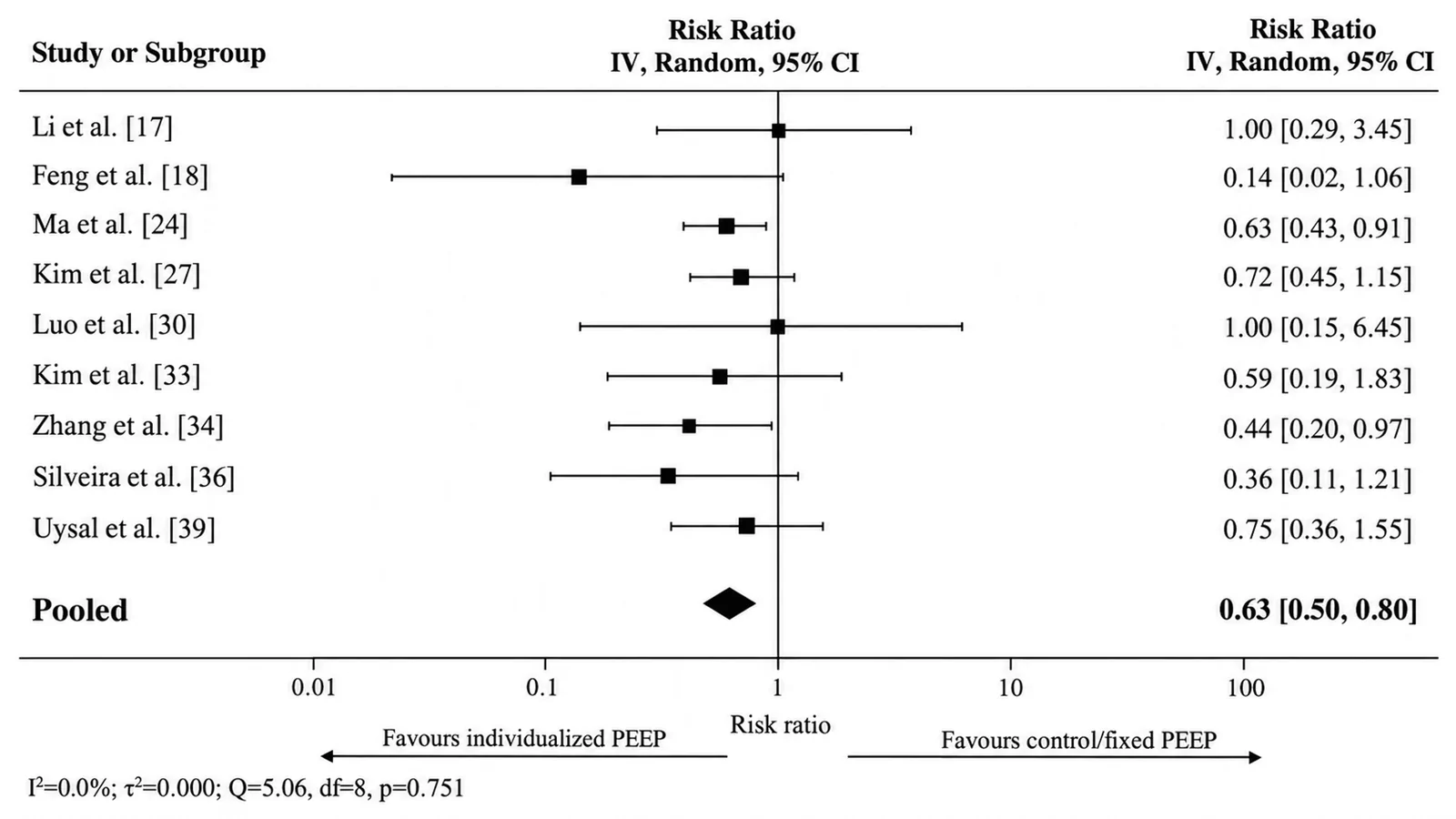
Random-effects meta-analysis of trial-defined postoperative pulmonary complications. Values below 1 favor individualized PEEP. Zhu et al. [37] had zero events in both arms and is described narratively but is not estimable in the RR model. Study marker colors distinguish rows only and do not represent subgroups. RR: risk ratio; CI: confidence interval; PEEP: positive end-expiratory pressure

Six studies contributed binary data for postoperative hypoxemia, desaturation, or clinically relevant oxygen requirement [19,24,26,27,30,35]. Events occurred in 58/333 participants receiving individualized PEEP and 108/339 receiving fixed or conventional PEEP. The pooled effect favored individualized PEEP (RR 0.52, 95% CI 0.30-0.89; P = 0.017), with moderate heterogeneity (I² = 57.9%; τ² = 0.216; Q = 11.88, df = 5, P = 0.036). These endpoints were clinically related but not identical, and they were assessed at different postoperative timepoints. Accordingly, the pooled estimate should be interpreted cautiously. The corresponding forest plot is shown in Figure 3.

**Figure 3:**
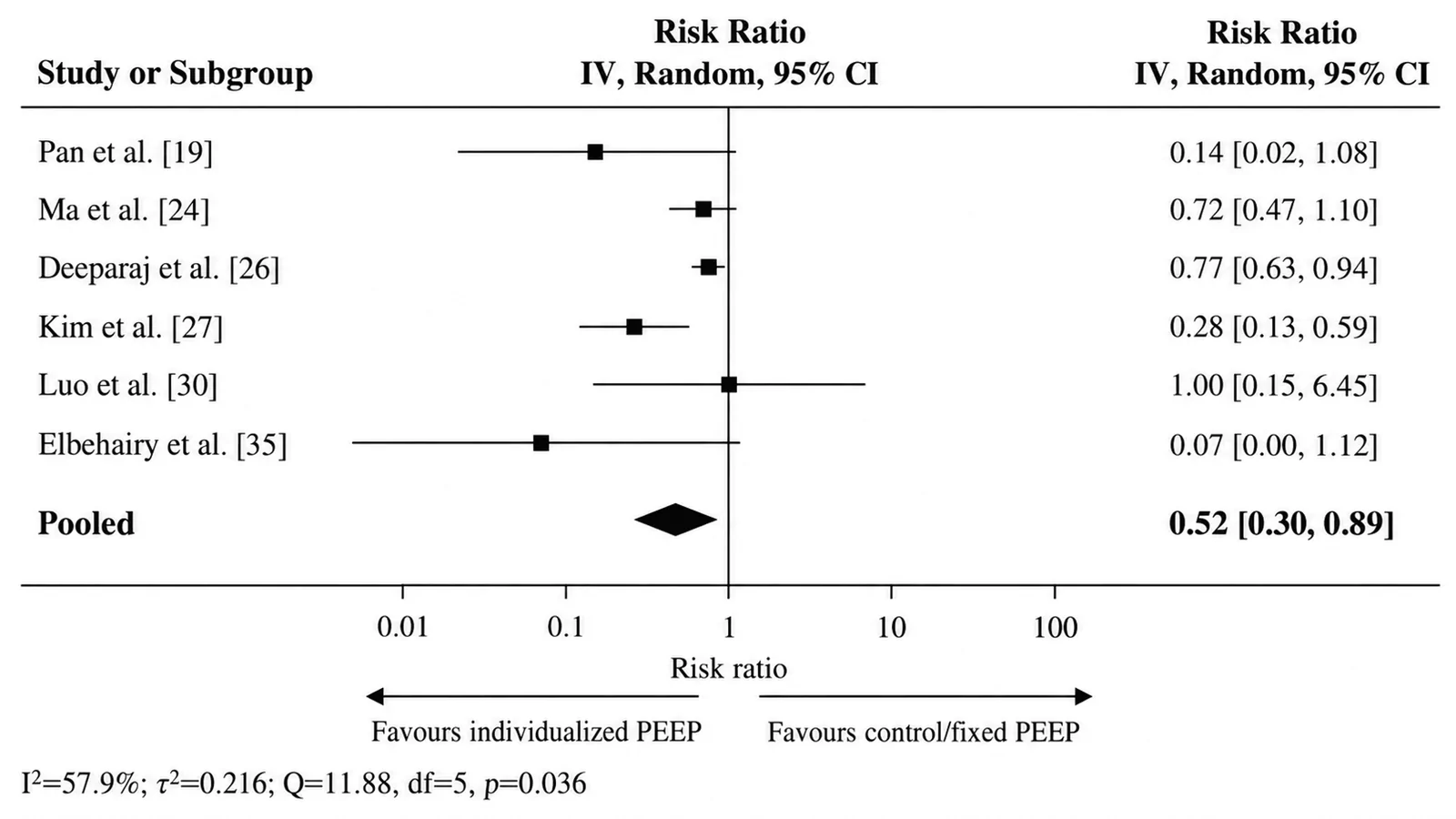
Random-effects meta-analysis of postoperative hypoxemia, desaturation, or clinically relevant oxygen requirement. Values below 1 favor individualized PEEP. Outcome definitions were clinically related but not identical, and the moderate heterogeneity should be considered when interpreting the pooled estimate. Study marker colors distinguish rows only and do not represent subgroups. CI: confidence interval; PEEP: positive end-expiratory pressure

Four trials provided compatible binary data for imaging- or lung-ultrasound-defined postoperative atelectasis [18,24,31,34]. Atelectasis occurred in 74/139 participants receiving individualized PEEP and 105/139 receiving fixed or conventional PEEP. The primary random-effects analysis favored individualized PEEP but narrowly crossed the null (RR 0.74, 95% CI 0.54-1.00; P = 0.051), with substantial heterogeneity (I² = 71.1%; τ² = 0.054; Q = 10.39, df = 3, P = 0.016). In a sensitivity analysis excluding Cho et al. [31], whose binary threshold was nearly universal despite lower continuous LUS, the pooled estimate was RR 0.64 (95% CI 0.48-0.87), with lower heterogeneity (I² = 38.8%). This sensitivity analysis was used to explore the influence of the threshold-sensitive study and does not replace the primary analysis. The primary binary atelectasis forest plot is shown in Figure 4.

**Figure 4:**
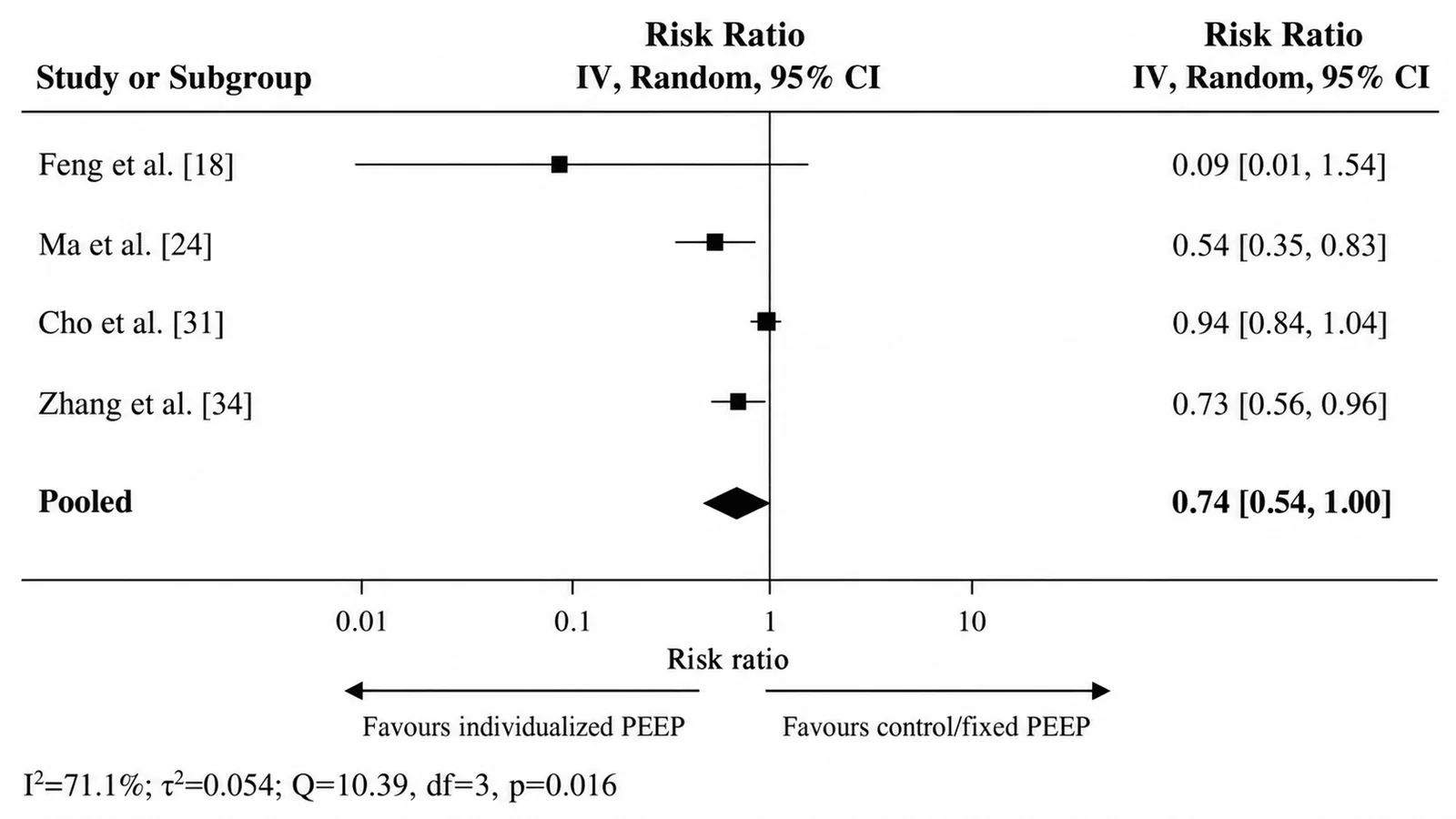
Random-effects meta-analysis of imaging- or lung-ultrasound-defined postoperative atelectasis. Values below 1 favor individualized PEEP. Definitions and postoperative assessment times varied. The primary analysis includes Cho et al. [31]; a sensitivity analysis excluding this threshold-sensitive study reduced heterogeneity. Study marker colors distinguish rows only and do not represent subgroups. CI: confidence interval; PEEP: positive end-expiratory pressure

Three trials reported sufficiently compatible early postoperative 12-zone total LUS values with arm-level means and standard deviations [25,32,39]. Across 194 participants, individualized PEEP reduced early postoperative LUS by a pooled MD of −3.09 points (95% CI −4.73 to −1.45; P < 0.001). Heterogeneity was moderate (I² = 51.3%; τ² = 1.088; Q = 4.11, df = 2, P = 0.128). Lower LUS indicates less postoperative loss of aeration. The forest plot is shown in Figure 5.

**Figure 5:**
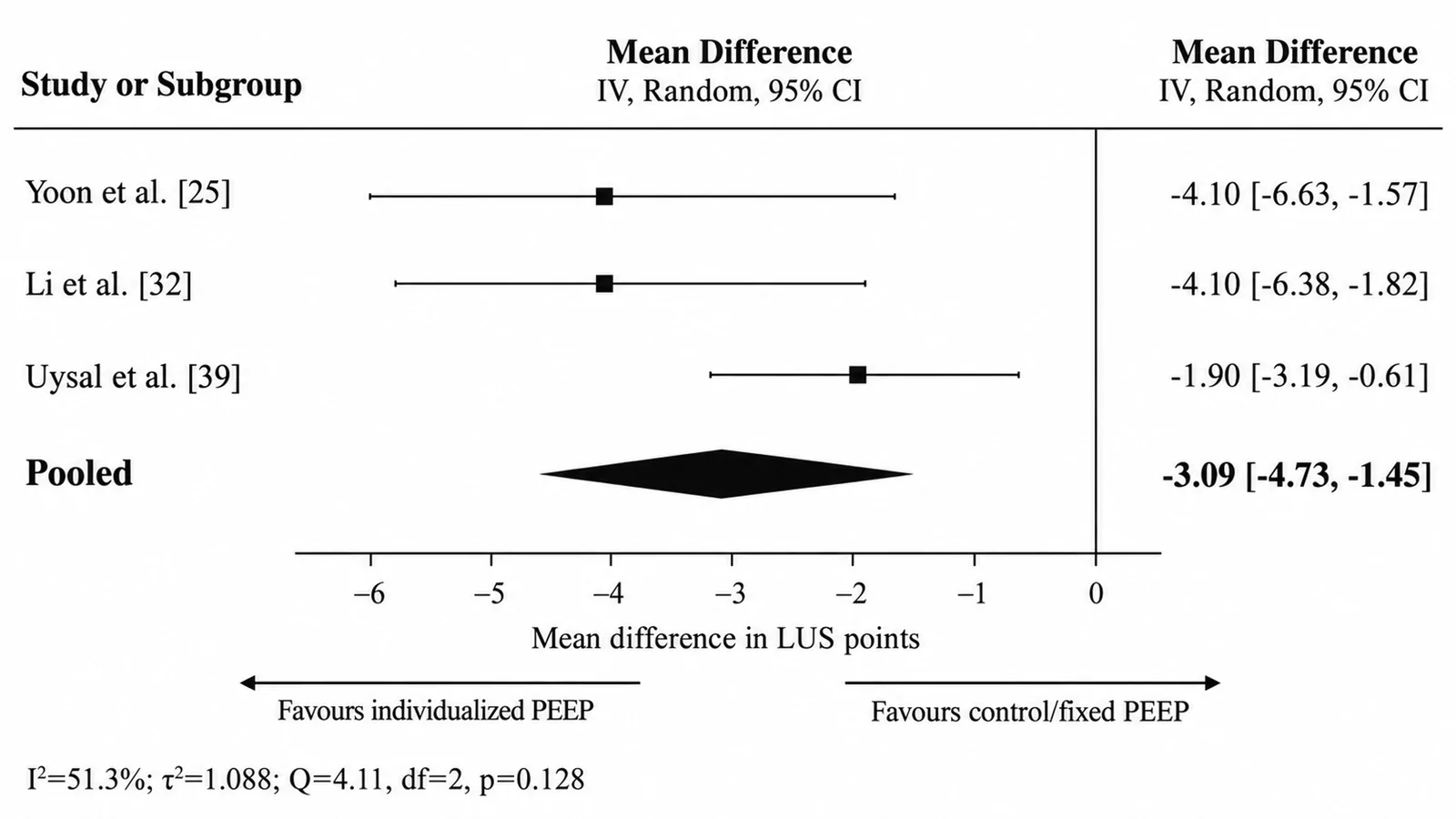
Random-effects meta-analysis of early postoperative 12-zone total LUS. Negative values favor individualized PEEP because lower LUS reflects less loss of aeration. Study marker colors distinguish rows only and do not represent subgroups. CI: confidence interval; LUS: lung-ultrasound score; PEEP: positive end-expiratory pressure

CT-derived percentage atelectasis, median/IQR lung-ultrasound outcomes, Clinical Pulmonary Infection Score, and several oxygenation measurements were retained for narrative synthesis because only one compatible study was available or because measurement scales and postoperative timepoints were not sufficiently comparable. Wang et al. [38] remained eligible but could not contribute numerical postoperative data to quantitative synthesis because arm-level postoperative results were not reported in an extractable form. The pooled analyses are summarized in Table 6.

**Table 6:** Summary of random-effects meta-analyses. Random-effects models used REML heterogeneity estimation. RR: risk ratio; MD: mean difference; CI: confidence interval; I²: proportion of observed variation attributable to heterogeneity; τ²: estimated between-study variance; PPC: postoperative pulmonary complication; LUS: lung-ultrasound score; REML: restricted maximum likelihood

Outcome	Studies (k)	Effect	Pooled estimate	95% CI	I²	τ²	Interpretation
PPCs	9	RR	0.63	0.50-0.80	0%	~0	Significant reduction; primary pooled analysis
Hypoxemia/desaturation/oxygen requirement	6	RR	0.52	0.30-0.89	57.9%	0.216	Significant reduction; moderate heterogeneity
Imaging-detected atelectasis	4	RR	0.74	0.54-1.00	71.1%	0.054	Borderline primary estimate; substantial heterogeneity
Early postoperative total LUS	3	MD	−3.09 points	−4.73 to −1.45	51.3%	1.088	Significantly lower LUS; moderate heterogeneity
Atelectasis sensitivity analysis excluding Cho et al. [31]	3	RR	0.64	0.48-0.87	38.8%	0.018	Significant reduction with lower heterogeneity

Risk-of-Bias Assessment

Risk of bias was assessed using the five domains of the Cochrane RoB 2 framework at the level of the principal postoperative result contributed by each trial. Five of 23 trials were judged to be at low overall risk of bias, and 18 raised some concerns; no trial was judged at high overall risk. Domain-level judgments are summarized in Table 7. Study-level judgments are displayed in Figure 6, and the distribution of judgments across domains is shown in Figure 7. Detailed study-level rationales are provided in Appendices A-E.

**Table 7:** Summary of RoB 2 judgments by domain. Percentages use the 23 included randomized trials as the denominator. Judgments refer to the principal postoperative outcome contributed by each trial. RoB 2: Risk of Bias 2; LUS: lung-ultrasound score

RoB 2 domain	Low risk, n (%)	Some concerns, n (%)	High risk, n (%)	Interpretation
Domain 1	7 (30.4%)	16 (69.6%)	0 (0.0%)	Concerns mainly reflected incomplete reporting of sequence generation or allocation concealment.
Domain 2	18 (78.3%)	5 (21.7%)	0 (0.0%)	Concerns mainly reflected post-randomization exclusions from the analyzed population.
Domain 3	17 (73.9%)	6 (26.1%)	0 (0.0%)	Concerns reflected incomplete outcome data or exclusions from the final analysis.
Domain 4	13 (56.5%)	10 (43.5%)	0 (0.0%)	Concerns mainly reflected uncertain assessor blinding for LUS or clinically assessed outcomes.
Domain 5	16 (69.6%)	7 (30.4%)	0 (0.0%)	Concerns mainly reflected unconfirmed prospective registration or prespecified analysis information.
Overall	5 (21.7%)	18 (78.3%)	0 (0.0%)	No study was judged at high overall risk; overall concerns primarily reflected reporting or verification limitations.

**Figure 6:**
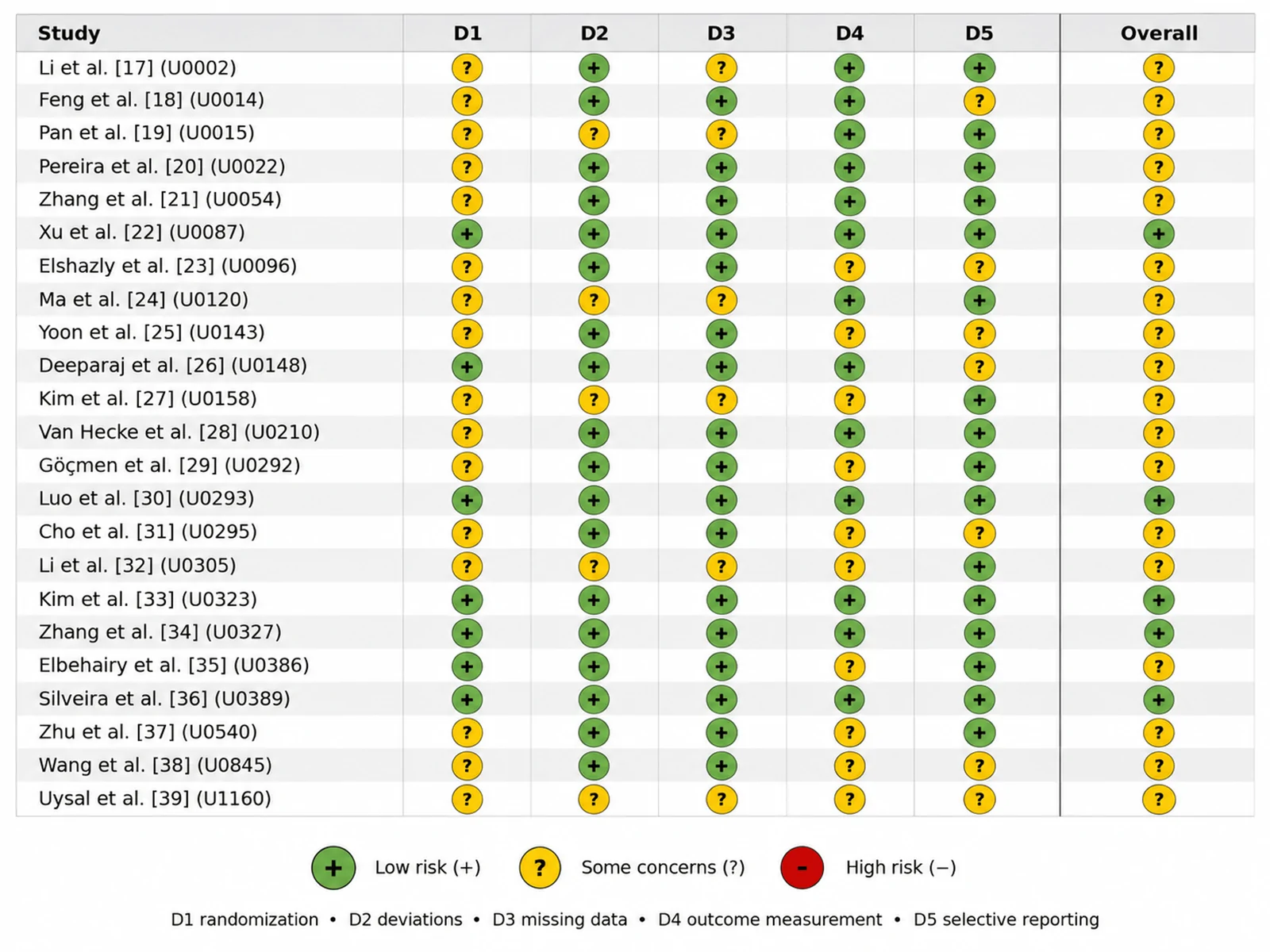
RoB 2 traffic-light summary for the principal postoperative outcome in each included study. Green (+) indicates low risk; yellow (?) indicates some concerns; red (−) indicates high risk. D1: randomization process; D2: deviations from intended interventions; D3: missing outcome data; D4: measurement of the outcome; D5: selection of the reported result; RoB 2: Risk of Bias 2

**Figure 7:**
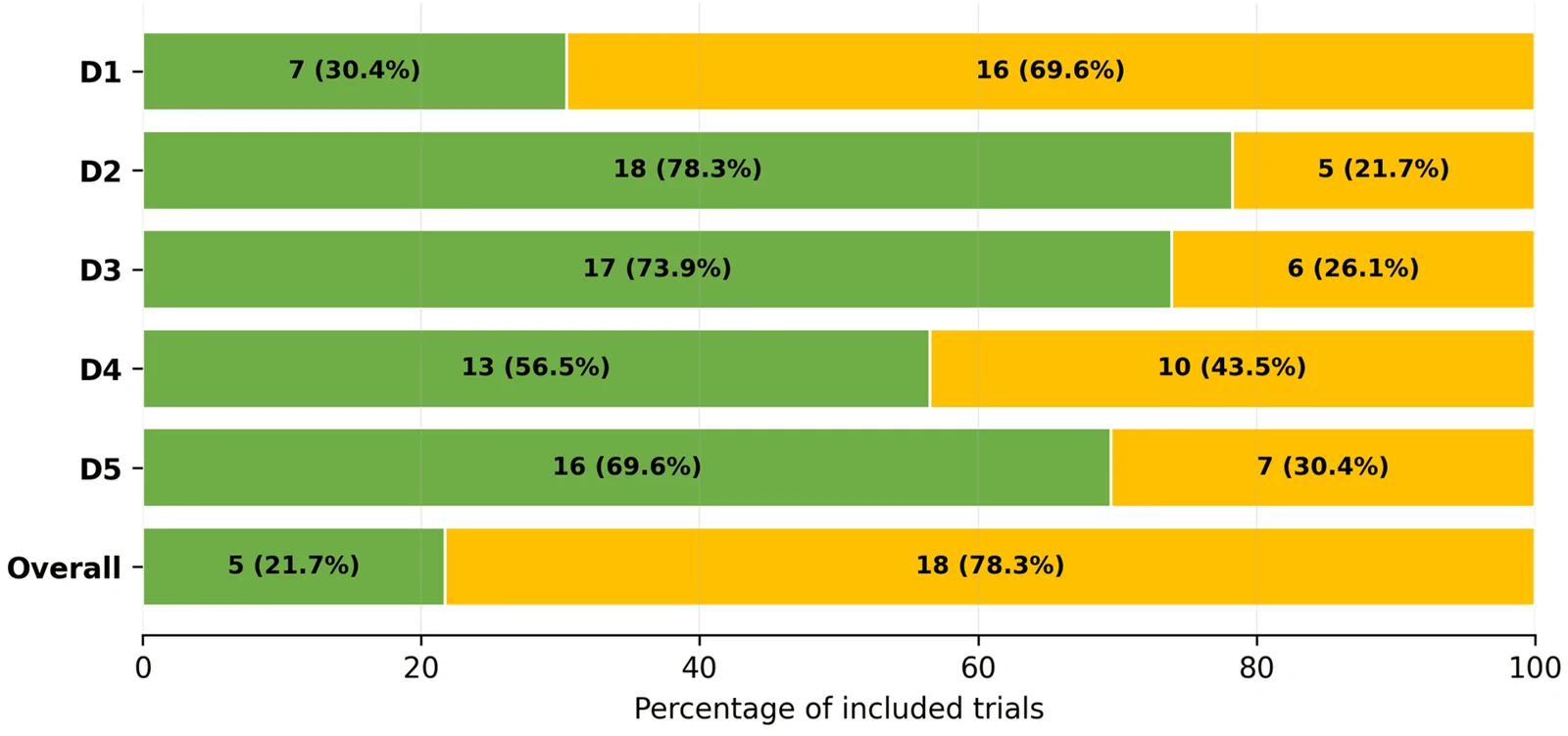
Distribution of RoB 2 judgments across domains and overall. Green indicates low risk of bias; yellow indicates some concerns; red indicates high risk of bias. Numbers within each bar represent the number and percentage of the 23 included trials assigned to each risk-of-bias category for that domain and for the overall judgment. D1: randomization process; D2: deviations from intended interventions; D3: missing outcome data; D4: measurement of the outcome; D5: selection of the reported result; RoB 2: Risk of Bias 2

Discussion

Principal Findings

This systematic review and meta-analysis of 23 randomized controlled trials found that individualized, titrated, or optimized intraoperative PEEP generally improved postoperative pulmonary outcomes compared with fixed or conventional PEEP in adults undergoing laparoscopic or robot-assisted abdominal-pelvic surgery. The strongest quantitative evidence was seen for composite PPCs, early postoperative hypoxemia or oxygen requirement, and early loss of lung aeration. Across nine estimable trials, individualized PEEP reduced PPCs with a pooled RR of 0.63 (95% CI 0.50-0.80; I² = 0%). Six compatible trials also showed fewer postoperative hypoxemia, desaturation, or oxygen-requirement events (RR 0.52, 95% CI 0.30-0.89), while three compatible studies reporting early total LUS showed less postoperative aeration loss (MD -3.09 points, 95% CI -4.73 to -1.45). These findings indicate that the benefit of individualized PEEP can extend beyond intraoperative respiratory mechanics to clinically relevant postoperative respiratory outcomes.

PPCs and Oxygenation

The reduction in PPCs was the most consistent clinical finding. PPCs occurred in 73 of 423 participants receiving individualized PEEP and 120 of 429 receiving fixed or conventional PEEP, corresponding to an approximately 37% relative reduction. Several trials showed clear numerical benefits, including Feng et al. [18], Ma et al. [24], Zhang et al. [34], and Silveira et al. [36], whereas the larger trial by Kim et al. [27] showed a directionally favorable but individually non-significant effect. Other smaller studies reported neutral or low-event results [17,30,33,37,39]. Importantly, these neutral findings did not suggest harm and were often limited by small sample size or few events. Although no statistical heterogeneity was detected in the pooled PPC analysis, this does not exclude clinical heterogeneity across studies. Differences in surgical procedures, PEEP-guidance methods, comparator PEEP levels, and PPC definitions remained clinically relevant and should be considered when interpreting the pooled estimate.

Postoperative oxygenation outcomes generally favored individualized PEEP, although the reported endpoints were clinically related rather than directly equivalent. Pan et al. [19] reported PACU hypoxemia in 1/26 participants receiving EIT-guided PEEP compared with 7/26 controls, while Kim et al. [27] reported fewer desaturation-related complications (8/178 vs. 30/185). Elbehairy et al. [35] reported postoperative hypoxia requiring supplemental oxygen in 0/30 participants in the individualized group compared with 7/30 controls, and Deeparaj et al. [26] found a lower need and shorter duration of postoperative oxygen supplementation. In contrast, Luo et al. [30] and Van Hecke et al. [28] showed little between-group difference. Uysal et al. [39] reported a higher PaO₂ during the first postoperative hour, but this difference was not maintained at later timepoints. Because these studies assessed different oxygenation-related endpoints and at different postoperative times, the pooled oxygenation findings should be interpreted cautiously. This clinical and temporal variability likely contributed to the moderate heterogeneity observed in the pooled analysis.

Atelectasis and Postoperative Lung Aeration

Direct measures of postoperative lung aeration were broadly favorable but were less uniform when converted into binary atelectasis outcomes. CT and lung-ultrasound studies generally showed less postoperative aeration loss with individualized PEEP [17,18,22,24-26,29,32,34,39]. The pooled analysis of three compatible early total lung-ultrasound studies showed a significant reduction in score, supporting better preservation of aeration. In contrast, four studies reporting binary atelectasis yielded a pooled RR of 0.74 (95% CI 0.54-1.00) with substantial heterogeneity (I² = 71.1%). Cho et al. [31] illustrate the reason for this discrepancy: continuous postoperative LUS were lower with individualized PEEP, yet the study-defined threshold for significant atelectasis was reached by nearly all participants in both groups. Excluding that trial in sensitivity analysis strengthened the pooled effect to RR 0.64 (95% CI 0.48-0.87). Overall, continuous imaging measures appear more sensitive to differences in postoperative aeration than study-specific binary thresholds.

Individualization Method and Clinical Context

No single PEEP-guidance method emerged as clearly superior. Favorable postoperative findings were reported with driving-pressure-, compliance-, EIT-, and lung-ultrasound-guided approaches [18-39], suggesting that the common therapeutic principle is patient-specific physiological selection rather than one particular monitoring technology. The importance of individualization may be greatest when respiratory mechanics change substantially, such as during obesity, pneumoperitoneum, or steep Trendelenburg positioning. Bariatric trials frequently required relatively high individualized PEEP levels [17,23,28,29,35,36], while robotic pelvic procedures showed favorable effects on mechanics or postoperative aeration despite less consistent differences in clinical PPCs [19,25,30,31,37-39]. Comparator PEEP also varied from approximately 4 to 10 cmH₂O, which may partly explain differences in effect size: a fixed PEEP of 4-5 cmH₂O may be clearly suboptimal in some patients, whereas a higher fixed comparator may approximate the physiological optimum for others. Recruitment maneuvers were another potential modifier, although the available data do not permit a reliable quantitative interaction analysis.

Interpretation of the Evidence

The findings should not be interpreted as evidence that universally higher PEEP is beneficial. The intervention evaluated across these trials was individualized PEEP, in which the level was selected according to a physiological or imaging response and therefore varied across patients and surgical phases. This distinction is clinically important because insufficient PEEP may permit derecruitment, whereas excessive PEEP may increase overdistension, driving pressure, or hemodynamic burden. The observed pattern is physiologically coherent: better intraoperative preservation of recruited lung regions may reduce postoperative aeration loss, which in turn may decrease early hypoxemia and contribute to fewer PPCs. The evidence therefore supports physiologically guided PEEP rather than routine assignment of the same high PEEP level to all patients.

Strengths and Limitations

This review has several strengths. Eligibility was restricted to randomized controlled trials and to laparoscopic or robot-assisted abdominal-pelvic surgery, avoiding the broader clinical heterogeneity created by combining open, thoracic, cardiac, and other surgical populations. Eligible studies were required to compare individualized PEEP with a fixed, conventional, or standard strategy and to report a postoperative pulmonary or direct lung-aeration outcome after extubation. Multiarm and mixed-population studies were handled conservatively, with recruitment-matched contrasts prioritized where possible and pooled open-plus-laparoscopic estimates excluded when the eligible minimally invasive subgroup could not be isolated. Clinical PPCs, binary atelectasis, continuous aeration measures, and hypoxemia-related outcomes were synthesized separately, and data that could not be extracted defensibly were retained in the narrative synthesis rather than reconstructed solely to increase the number of studies in meta-analysis.

Several limitations remain. Most trials were small and single-center, limiting power for uncommon severe outcomes such as pneumonia, respiratory failure, reintubation, or unplanned postoperative ventilation. Clinical heterogeneity persisted across surgical populations, baseline pulmonary risk, PEEP-selection methods, comparator levels, recruitment strategies, and assessment times. Outcome definitions were particularly variable for atelectasis and hypoxemia, which likely contributed to the higher heterogeneity in those analyses. Some studies reported medians, graph-only findings, or insufficient arm-level numerical data and therefore could not enter quantitative synthesis without additional assumptions [20,38]. Risk-of-bias assessment identified some concerns in a substantial proportion of the evidence base, commonly related to incomplete reporting of allocation concealment, post-randomization exclusions, assessor blinding, or prespecified outcome reporting. Finally, the number of compatible studies within individual guidance methods or surgical subgroups was too small for definitive subgroup-effect conclusions.

Clinical Implications and Future Research

For clinical practice, the evidence suggests that individualized PEEP is preferable to routinely assigning the same PEEP level to all patients when postoperative pulmonary risk is important, particularly in settings with major changes in respiratory mechanics such as obesity, pneumoperitoneum, and steep Trendelenburg positioning. The strongest evidence relates to fewer composite PPCs, fewer early oxygenation problems, and better preservation of postoperative lung aeration. However, the data do not establish one universally superior titration method; driving pressure, compliance, EIT, and lung ultrasound all produced favorable results in at least some randomized trials. Selection of method should therefore reflect local expertise, available monitoring, and procedural context, and retitration may be reasonable after major physiological changes during surgery.

Future trials should be larger, multicenter, and powered for clinically important postoperative pulmonary endpoints rather than primarily intraoperative physiology. Standardized definitions of PPCs, atelectasis, hypoxemia, lung-ultrasound scoring, and postoperative assessment times would improve comparability and reduce avoidable heterogeneity. Direct comparisons of individualized-PEEP guidance methods, while retaining a valid fixed-PEEP reference arm, are needed to determine whether one approach offers an advantage. Further research should also establish whether individualized PEEP reduces pneumonia, respiratory failure, intensive-care use, length of stay, or unplanned postoperative ventilation, and whether treatment effects differ according to obesity, baseline pulmonary risk, robotic versus conventional laparoscopy, degree of Trendelenburg positioning, or recruitment-maneuver strategy.

## Conclusions

Current evidence suggests that individualized intraoperative PEEP may improve postoperative pulmonary outcomes compared with fixed or conventional PEEP in adults undergoing laparoscopic or robot-assisted abdominal-pelvic surgery. The most consistent findings were fewer composite pulmonary complications, fewer early oxygenation problems, and better postoperative lung aeration, whereas binary atelectasis outcomes were more heterogeneous. Benefits were observed across several physiological and imaging-guided titration approaches, supporting patient-specific rather than universal fixed PEEP selection.

However, differences in patient populations, titration methods, diagnostic definitions, and postoperative assessment times limit certainty about the optimal personalization strategy. Larger multicenter trials using standardized postoperative pulmonary endpoints are needed to determine which titration methods and patient groups derive the greatest clinical benefit.

## Appendices

Supplementary detailed risk-of-bias tables

Appendices A-E provide the detailed study-level RoB 2 judgments and supporting rationale corresponding to the summary presented in Table 7 and Figures 6, 7.

Appendix A

**Table 8:** RoB 2 domain 1 - bias arising from the randomization process. Judgments are result-level assessments for the principal postoperative outcome. RoB 2: Risk of Bias 2

Study ID/citation	Judgment	Rationale
Li et al. [17] (U0002)	Some concerns	Randomization was reported, but sequence-generation or allocation-concealment details were incompletely documented in the published report.
Feng et al. [18] (U0014)	Some concerns	Computer-generated randomization with sealed envelopes was reported, but registry timing followed the start of enrollment.
Pan et al. [19] (U0015)	Some concerns	Randomization was reported, but sequence-generation or allocation-concealment details were incompletely documented in the published report.
Pereira et al. [20] (U0022)	Some concerns	Randomization was reported, but sequence-generation or allocation-concealment details were incompletely documented in the published report.
Zhang et al. [21] (U0054)	Some concerns	Randomization was reported, but sequence-generation or allocation-concealment details were incompletely documented in the published report.
Xu et al. [22] (U0087)	Low risk	Adequate random-sequence generation and allocation concealment were reported or verified.
Elshazly et al. [23] (U0096)	Some concerns	Randomization was reported, but sequence-generation or allocation-concealment details were incompletely documented in the published report.
Ma et al. [24] (U0120)	Some concerns	Randomization was reported, but sequence-generation or allocation-concealment details were incompletely documented in the published report.
Yoon et al. [25] (U0143)	Some concerns	Randomization was reported, but sequence-generation or allocation-concealment details were incompletely documented in the published report.
Deeparaj et al. [26] (U0148)	Low risk	Computer-generated random-number table and sealed opaque envelopes were reported.
Kim et al. [27] (U0158)	Some concerns	Randomization was reported, but sequence-generation or allocation-concealment details were incompletely documented in the published report.
Van Hecke et al. [28] (U0210)	Some concerns	Randomization was reported, but sequence-generation or allocation-concealment details were incompletely documented in the published report.
Göçmen et al. [29] (U0292)	Some concerns	Randomization was reported, but sequence-generation or allocation-concealment details were incompletely documented in the published report.
Luo et al. [30] (U0293)	Low risk	Computer-generated sequence and opaque sealed-envelope allocation were reported.
Cho et al. [31] (U0295)	Some concerns	Randomization was reported, but sequence-generation or allocation-concealment details were incompletely documented in the published report.
Li et al. [32] (U0305)	Some concerns	Randomization was reported, but sequence-generation or allocation-concealment details were incompletely documented in the published report.
Kim et al. [33] (U0323)	Low risk	Independent computer-generated sequence and opaque-envelope concealment were reported.
Zhang et al. [34] (U0327)	Low risk	Computer-generated random list and sequential opaque sealed envelopes were reported.
Elbehairy et al. [35] (U0386)	Low risk	Computer-generated assignments and sealed opaque envelopes were reported.
Silveira et al. [36] (U0389)	Low risk	Independent computer-generated permuted blocks and sequential opaque envelopes were reported.
Zhu et al. [37] (U0540)	Some concerns	Randomization was reported, but sequence-generation or allocation-concealment details were incompletely documented in the published report.
Wang et al. [38] (U0845)	Some concerns	Randomization was reported, but sequence-generation or allocation-concealment details were incompletely documented in the published report.
Uysal et al. [39] (U1160)	Some concerns	Randomization was reported, but sequence-generation or allocation-concealment details were incompletely documented in the published report.

Appendix B

**Table 9:** RoB 2 domain 2 - bias due to deviations from intended interventions. Judgments are result-level assessments for the principal postoperative outcome. RoB 2: Risk of Bias 2

Study ID/citation	Judgment	Rationale
Li et al. [17] (U0002)	Low risk	No important deviations from the assigned ventilation strategy likely to bias the treatment-effect estimate were identified.
Feng et al. [18] (U0014)	Low risk	No important deviations from the assigned ventilation strategy likely to bias the treatment-effect estimate were identified.
Pan et al. [19] (U0015)	Some concerns	Four of 56 randomized participants were excluded from the final analysis.
Pereira et al. [20] (U0022)	Low risk	No important deviations from the assigned ventilation strategy likely to bias the treatment-effect estimate were identified.
Zhang et al. [21] (U0054)	Low risk	No important deviations from the assigned ventilation strategy likely to bias the treatment-effect estimate were identified.
Xu et al. [22] (U0087)	Low risk	No important deviations from the assigned ventilation strategy likely to bias the treatment-effect estimate were identified.
Elshazly et al. [23] (U0096)	Low risk	No important deviations from the assigned ventilation strategy likely to bias the treatment-effect estimate were identified.
Ma et al. [24] (U0120)	Some concerns	Seven of 80 randomized participants were excluded from the final analysis.
Yoon et al. [25] (U0143)	Low risk	No important deviations from the assigned ventilation strategy likely to bias the treatment-effect estimate were identified.
Deeparaj et al. [26] (U0148)	Low risk	No important deviations from the assigned ventilation strategy likely to bias the treatment-effect estimate were identified.
Kim et al. [27] (U0158)	Some concerns	Twenty-one of 384 randomized participants were not included in the analyzed population.
Van Hecke et al. [28] (U0210)	Low risk	No important deviations from the assigned ventilation strategy likely to bias the treatment-effect estimate were identified.
Göçmen et al. [29] (U0292)	Low risk	No important deviations from the assigned ventilation strategy likely to bias the treatment-effect estimate were identified.
Luo et al. [30] (U0293)	Low risk	No important deviations from the assigned ventilation strategy likely to bias the treatment-effect estimate were identified.
Cho et al. [31] (U0295)	Low risk	No important deviations from the assigned ventilation strategy likely to bias the treatment-effect estimate were identified.
Li et al. [32] (U0305)	Some concerns	Six of 70 randomized participants were excluded from the final laparoscopic analysis.
Kim et al. [33] (U0323)	Low risk	No important deviations from the assigned ventilation strategy likely to bias the treatment-effect estimate were identified.
Zhang et al. [34] (U0327)	Low risk	No important deviations from the assigned ventilation strategy likely to bias the treatment-effect estimate were identified.
Elbehairy et al. [35] (U0386)	Low risk	No important deviations from the assigned ventilation strategy likely to bias the treatment-effect estimate were identified.
Silveira et al. [36] (U0389)	Low risk	No important deviations from the assigned ventilation strategy likely to bias the treatment-effect estimate were identified.
Zhu et al. [37] (U0540)	Low risk	No important deviations from the assigned ventilation strategy likely to bias the treatment-effect estimate were identified.
Wang et al. [38] (U0845)	Low risk	No important deviations from the assigned ventilation strategy likely to bias the treatment-effect estimate were identified.
Uysal et al. [39] (U1160)	Some concerns	Six of 76 randomized participants were excluded, including surgical conversions/withdrawal.

Appendix C 

**Table 10:** RoB 2 domain 3 - bias due to missing outcome data. Judgments are result-level assessments for the principal postoperative outcome. RoB 2: Risk of Bias 2; CT: computed tomography

Study ID/citation	Judgment	Rationale
Li et al. [17] (U0002)	Some concerns	Postoperative CT data were available for 19/20 participants per arm.
Feng et al. [18] (U0014)	Low risk	Outcome data were complete or missingness was small and unlikely to materially bias the principal postoperative result.
Pan et al. [19] (U0015)	Some concerns	52/56 randomized participants were included in the final analysis.
Pereira et al. [20] (U0022)	Low risk	Outcome data were complete or missingness was small and unlikely to materially bias the principal postoperative result.
Zhang et al. [21] (U0054)	Low risk	Outcome data were complete or missingness was small and unlikely to materially bias the principal postoperative result.
Xu et al. [22] (U0087)	Low risk	Outcome data were complete or missingness was small and unlikely to materially bias the principal postoperative result.
Elshazly et al. [23] (U0096)	Low risk	Outcome data were complete or missingness was small and unlikely to materially bias the principal postoperative result.
Ma et al. [24] (U0120)	Some concerns	73/80 randomized participants were included in the final analysis.
Yoon et al. [25] (U0143)	Low risk	Outcome data were complete or missingness was small and unlikely to materially bias the principal postoperative result.
Deeparaj et al. [26] (U0148)	Low risk	Outcome data were complete or missingness was small and unlikely to materially bias the principal postoperative result.
Kim et al. [27] (U0158)	Some concerns	363/384 randomized participants were analyzed.
Van Hecke et al. [28] (U0210)	Low risk	Outcome data were complete or missingness was small and unlikely to materially bias the principal postoperative result.
Göçmen et al. [29] (U0292)	Low risk	Outcome data were complete or missingness was small and unlikely to materially bias the principal postoperative result.
Luo et al. [30] (U0293)	Low risk	Outcome data were complete or missingness was small and unlikely to materially bias the principal postoperative result.
Cho et al. [31] (U0295)	Low risk	Outcome data were complete or missingness was small and unlikely to materially bias the principal postoperative result.
Li et al. [32] (U0305)	Some concerns	64/70 randomized participants were included in the final laparoscopic analysis.
Kim et al. [33] (U0323)	Low risk	Outcome data were complete or missingness was small and unlikely to materially bias the principal postoperative result.
Zhang et al. [34] (U0327)	Low risk	Outcome data were complete or missingness was small and unlikely to materially bias the principal postoperative result.
Elbehairy et al. [35] (U0386)	Low risk	Outcome data were complete or missingness was small and unlikely to materially bias the principal postoperative result.
Silveira et al. [36] (U0389)	Low risk	Outcome data were complete or missingness was small and unlikely to materially bias the principal postoperative result.
Zhu et al. [37] (U0540)	Low risk	Outcome data were complete or missingness was small and unlikely to materially bias the principal postoperative result.
Wang et al. [38] (U0845)	Low risk	Outcome data were complete or missingness was small and unlikely to materially bias the principal postoperative result.
Uysal et al. [39] (U1160)	Some concerns	70/76 randomized participants were included in the final analysis.

Appendix D

**Table 11:** RoB 2 domain 4 - bias in measurement of the outcome. Judgments are result-level assessments for the principal postoperative outcome. RoB 2: Risk of Bias 2; PPC: postoperative pulmonary complication

Study ID/citation	Judgment	Rationale
Li et al. [17] (U0002)	Low risk	The principal outcome was objective or protocolized, with no clear evidence of differential measurement between groups.
Feng et al. [18] (U0014)	Low risk	The principal outcome was objective or protocolized, with no clear evidence of differential measurement between groups.
Pan et al. [19] (U0015)	Low risk	The principal outcome was objective or protocolized, with no clear evidence of differential measurement between groups.
Pereira et al. [20] (U0022)	Low risk	The principal outcome was objective or protocolized, with no clear evidence of differential measurement between groups.
Zhang et al. [21] (U0054)	Low risk	The principal outcome was objective or protocolized, with no clear evidence of differential measurement between groups.
Xu et al. [22] (U0087)	Low risk	The principal outcome was objective or protocolized, with no clear evidence of differential measurement between groups.
Elshazly et al. [23] (U0096)	Some concerns	Postoperative hypoxia/chest-radiographic collapse was clinically assessed; blinding of assessors was not clearly documented.
Ma et al. [24] (U0120)	Low risk	The principal outcome was objective or protocolized, with no clear evidence of differential measurement between groups.
Yoon et al. [25] (U0143)	Some concerns	Lung ultrasound was the principal endpoint, but assessor blinding was not fully verified.
Deeparaj et al. [26] (U0148)	Low risk	The principal outcome was objective or protocolized, with no clear evidence of differential measurement between groups.
Kim et al. [27] (U0158)	Some concerns	The PPC composite included clinical components; blinded outcome adjudication was not fully verified.
Van Hecke et al. [28] (U0210)	Low risk	The principal outcome was objective or protocolized, with no clear evidence of differential measurement between groups.
Göçmen et al. [29] (U0292)	Some concerns	Modified lung-ultrasound scoring was used, but assessor blinding was not fully established.
Luo et al. [30] (U0293)	Low risk	The principal outcome was objective or protocolized, with no clear evidence of differential measurement between groups.
Cho et al. [31] (U0295)	Some concerns	Postoperative lung ultrasound was central to the endpoint; assessor blinding was not fully verified.
Li et al. [32] (U0305)	Some concerns	Postoperative lung-ultrasound scoring was used; assessor blinding was not fully verified.
Kim et al. [33] (U0323)	Low risk	The principal outcome was objective or protocolized, with no clear evidence of differential measurement between groups.
Zhang et al. [34] (U0327)	Low risk	The principal outcome was objective or protocolized, with no clear evidence of differential measurement between groups.
Elbehairy et al. [35] (U0386)	Some concerns	Postoperative hypoxia requiring oxygen may involve clinical decision-making; blinded ascertainment was not fully documented.
Silveira et al. [36] (U0389)	Low risk	The principal outcome was objective or protocolized, with no clear evidence of differential measurement between groups.
Zhu et al. [37] (U0540)	Some concerns	Clinical PPC criteria/chest imaging were used; blinding of assessors was not fully documented.
Wang et al. [38] (U0845)	Some concerns	The postoperative aeration/PPC assessment process could not be fully appraised from the published report.
Uysal et al. [39] (U1160)	Some concerns	Lung-ultrasound assessment was investigator-performed and blinding to randomized strategy was not explicitly documented.

Appendix E 

**Table 12:** RoB 2 domain 5 - bias in selection of the reported result. Judgments are result-level assessments for the principal postoperative outcome. RoB 2: Risk of Bias 2

Study ID/citation	Judgment	Rationale
Li et al. [17] (U0002)	Low risk	A trial registration/protocol was available and the principal postoperative outcome used in this review was reported.
Feng et al. [18] (U0014)	Some concerns	The trial was registered, but registry timing followed the start of participant enrollment.
Pan et al. [19] (U0015)	Low risk	A trial registration/protocol was available and the principal postoperative outcome used in this review was reported.
Pereira et al. [20] (U0022)	Low risk	A trial registration/protocol was available and the principal postoperative outcome used in this review was reported.
Zhang et al. [21] (U0054)	Low risk	A trial registration/protocol was available and the principal postoperative outcome used in this review was reported.
Xu et al. [22] (U0087)	Low risk	A trial registration/protocol was available and the principal postoperative outcome used in this review was reported.
Elshazly et al. [23] (U0096)	Some concerns	Prospective registration or a sufficiently detailed prespecified analysis record was not confirmed in the published report.
Ma et al. [24] (U0120)	Low risk	A trial registration/protocol was available and the principal postoperative outcome used in this review was reported.
Yoon et al. [25] (U0143)	Some concerns	Prospective registration or a sufficiently detailed prespecified analysis record was not confirmed in the published report.
Deeparaj et al. [26] (U0148)	Some concerns	Prospective registration or a sufficiently detailed prespecified analysis record was not confirmed in the published report.
Kim et al. [27] (U0158)	Low risk	A trial registration/protocol was available and the principal postoperative outcome used in this review was reported.
Van Hecke et al. [28] (U0210)	Low risk	A trial registration/protocol was available and the principal postoperative outcome used in this review was reported.
Göçmen et al. [29] (U0292)	Low risk	A trial registration/protocol was available and the principal postoperative outcome used in this review was reported.
Luo et al. [30] (U0293)	Low risk	A trial registration/protocol was available and the principal postoperative outcome used in this review was reported.
Cho et al. [31] (U0295)	Some concerns	Prospective registration or a sufficiently detailed prespecified analysis record was not confirmed in the published report.
Li et al. [32] (U0305)	Low risk	A trial registration/protocol was available and the principal postoperative outcome used in this review was reported.
Kim et al. [33] (U0323)	Low risk	A trial registration/protocol was available and the principal postoperative outcome used in this review was reported.
Zhang et al. [34] (U0327)	Low risk	A trial registration/protocol was available and the principal postoperative outcome used in this review was reported.
Elbehairy et al. [35] (U0386)	Low risk	A trial registration/protocol was available and the principal postoperative outcome used in this review was reported.
Silveira et al. [36] (U0389)	Low risk	A trial registration/protocol was available and the principal postoperative outcome used in this review was reported.
Zhu et al. [37] (U0540)	Low risk	A trial registration/protocol was available and the principal postoperative outcome used in this review was reported.
Wang et al. [38] (U0845)	Some concerns	No trial registration was confirmed and numerical postoperative results were incompletely accessible for verification.
Uysal et al. [39] (U1160)	Some concerns	Prospective registration or a sufficiently detailed prespecified analysis record was not confirmed in the published report.
